# Management of Asbestos Containing Materials: A Detailed LCA Comparison of Different Scenarios Comprising First Time Asbestos Characterization Factor Proposal

**DOI:** 10.1021/acs.est.1c02410

**Published:** 2021-09-01

**Authors:** Martina Pini, Simone Scarpellini, Roberto Rosa, Paolo Neri, Alessandro F. Gualtieri, Anna Maria Ferrari

**Affiliations:** † Department of Sciences and Methods for Engineering, University of Modena and Reggio Emilia, Via G. Amendola 2, 42122, Reggio Emilia, Italy; ‡ Interdepartmental Center En&Tech, University of Modena and Reggio Emilia, Via G. Amendola, 2, 42122 Reggio Emilia, Italy; § Department of Chemical and Geological Sciences, University of Modena and Reggio Emilia, Via G. Campi 103, 41125, Modena, Italy

**Keywords:** life cycle assessment, environmental sustainability, asbestos containing waste, landfilling, thermal inertisation

## Abstract

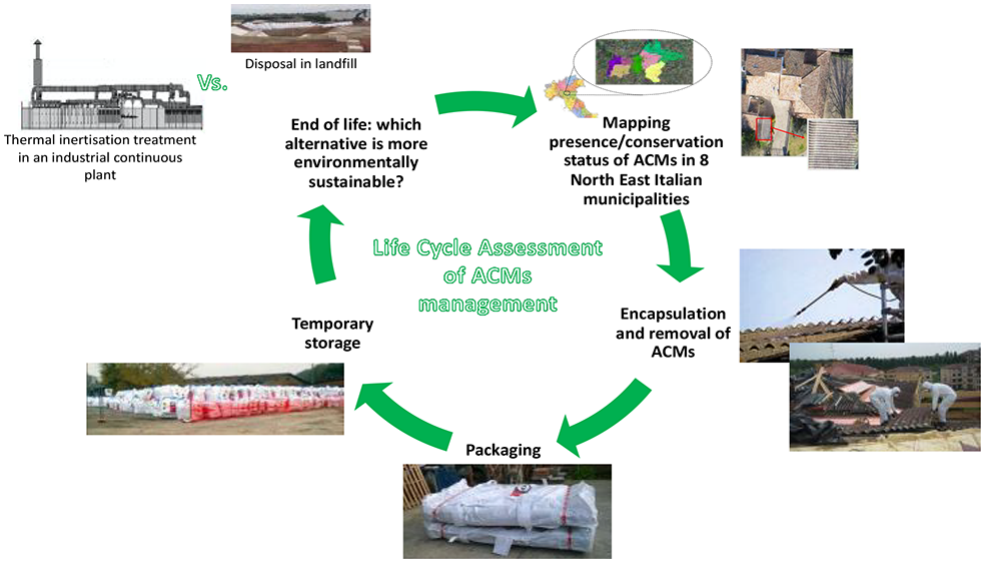

This work addresses the complex issue of asbestos containing materials (ACMs) management, by focusing on the scenario of six municipalities comprised in the Reggio Emilia province of Emilia Romagna Italian region. Particularly, the life cycle assessment (LCA) methodology was applied in order to assess in a quantitative and reliable manner the human toxicity as well as the ecotoxicity impacts associated with all of the different phases of ACMs management. The latter comprises mapping of ACMs, creation of a risk map for defining priority of intervention, encapsulation and removal of ACMs, as well as the as obtained asbestos containing waste (ACW) end of life. Particularly, a thermal inertisation treatment performed in a continuous industrial furnace was considered as the innovative end of life scenario to be compared with what actually was provided by the legislation of many countries worldwide, that is, the disposal of ACW in a controlled landfill for hazardous wastes. A characterization factor for asbestos fibers released both in outdoor air and in occupational setting was proposed for the first time and included in the USEtox 2.0 impact assessment method. This allowed us to reliably and quantitatively highlight that inertisation treatments should be the preferred solutions to be adopted by local and national authorities, especially if the obtained inert material finds application as secondary raw materials, thus contributing to a decrease in the environmental damage (limited to its toxicological contributions) to be associated with asbestos management.

## Abstract 2

Asbestos characterization factor was developed to reliably compare the human and ecotoxicological impacts of ACMs management considering different end of life scenarios.

## Introduction

Asbestos, a commercial term referring to six different silicate minerals (i.e., chrysotile, actinolite asbestos, amosite, anthophyllite asbestos, crocidolite, and tremolite asbestos^1^), has been used as a building material since ancient times for its outstanding physical–chemical and technological properties.^2,3^ Although the above-mentioned properties arise from its peculiar crystal habit (i.e., fibrous asbestiform), the latter is also responsible for severe health hazard, including pulmonary asbestosis, malignant mesothelioma and lung cancer,^3−5^ so that asbestos has been included in Group 1 of carcinogens (i.e., carcinogenic for humans) by the IARC (International Agency for Research on Cancer).^6^

Since the early 1970s, many countries started banning the production of asbestos containing materials (ACMs). Italy, for example, definitively banned asbestos in 1992 with the Italian legislative decree no. 257/92.^7^ However, due to its widespread utilization, huge amounts of ACMs still remain present in both private and public buildings,^8^ so that to avoid any possible human health and environmental risks associated with inhalation of asbestos fibers as a consequence for example of catastrophic events or the natural aging/decomposition of these ACMs, many communities prompted the development of opportune plans for the safe removal of ACMs.^9^ According to the actual regulation of different countries worldwide, the as-obtained asbestos containing waste (ACW) is then typically dumped in controlled landfills, only postponing the environmental and human health issues to the future generations.^10^ In this way, however, the absence of asbestos fibers release in the atmosphere and in hydrologic systems (as a consequence of the possible action of acid-corrosive agents in the leachate) cannot be guaranteed. For this reason, several asbestos inertisation treatments have been proposed in the last decades, most of which has been summarized in a recent review.^11^ Particularly, they include chemical,^12^ thermal,^13,14^ thermochemical^15,16^ and mechanical procedures,^17−19^ together with those based on vitrification^20^ as well as biological treatments.^21^

However, although the European Union recently recognized asbestos inertisation as a preferable solution to be adopted instead of landfilling in order to decrease the environmental burdens associated with ACW,^22^ most of the proposed methodologies are extremely energy demanding, or not yet sufficiently mature,^11^ thus risking to simply move the environmental impacts to a different phase of the asbestos life cycle. Therefore, reliable quantitative environmental assessments of different scenarios for ACW are necessary to identify in a trustworthy manner the less environmentally impacting solution, considering its whole life cycle. Life cycle assessment (LCA) methodology represents a standardized environmental management tool to quantify the potential environmental impacts associated with a process or product during its whole life cycle.^23−25^ However, the LCA studies applied to the different management possibilities for asbestos containing waste, are surprisingly infrequent,^26^ and this is due to the fact that no impact assessment method exists for asbestos emissions in soil, water, or air.^27^

Some strategies have been proposed to partially overcome this limitation. The work by Terazono et al.,^26^ although limitedly to the disposal stage of asbestos life cycle, for the first time attempted at quantifying the health risk of asbestos, by proposing a solution to the pulse-flux issue (i.e., the difficulty in calculating the health risk from an emission due to the lack of data related to emission duration), through an estimation of asbestos exposure dose and the calculation of the conversion factor expressing the relationship between exposure dose and health risk. The different environmental issues considered in that study (i.e., health risk and energy consumption) were not weighted since, at that time, transparent and fair methods of weighting, for comparing their effects were yet missing in most of the impact assessment methods used in LCA. More recently, Loss et al.^27^ introduced inventory indicators accounting for the air dispersion of asbestos fibers as well as for underground deposition of asbestos. However, it was only possible to quantify the entity of these emission and deposits on a mere mass base, since again, the impact assessment method used in that study (i.e., ReCiPe 2008 H/H Europe^28^) is not able to associate environmental impact to asbestos.

Oppositely, the present paper proposes a first attempt to calculate the human health characterization factor for asbestos fibers for both outdoor and indoor air environments, by applying USEtox 2.0 consensus model,^29^ to make human and ecotoxicological impacts assessment of different scenarios for ACW treatment/end of life, comparable at all and on a more reliable basis. Similar approaches have been recently applied in order to assess freshwater ecotoxicity and human toxicity associated with TiO_2_ nanoparticles^30^ and perfluoroalkyl acids.^31^

This work focuses on the management of ACMs in the coverings of private and public buildings in the eight municipalities of the “Unione dei Comuni della Bassa Reggiana” (Reggio Emilia province, Emilia Romagna region, Italy).^32^ The study includes the mapping activity, the creation of a risk map for defining the priority of interventions, and all the activities performed for encapsulation and safe removal of ACMs. Two treatment scenarios for ACW were considered and quantitatively compared from the environmental perspective: an innovative thermal inertisation treatment employing an industrial continuous furnace, as recently patented by some of the present authors,^33^ and what actually provided by the Italian legislation, that is, ACW disposal in controlled landfills.

The inert material obtained by the thermal inertisation treatment was considered a coproduct of the process, thus a secondary raw material, usable in the production of porcelain stoneware slabs, as recently demonstrated.^34^ A further comparison was made with the scenario characterized by the lack of any ACMs removal actions, in order to highlight the necessity and urgency of intervention, mainly from the human toxicity perspective.

This work allows for the first time to establish in a quantitative manner the management solution for ACMs characterized by the lower impacts on human toxicity and freshwater ecotoxicity (among the investigated alternatives), by considering their whole life cycle. Moreover, the proposed asbestos characterization factor, will smooth the way toward highly desirable LCA studies referred to further management scenarios, with the aim to guide and support future decisions making by local and national authorities.

## Experimental Details of the ACMs Management Phases Assessed

### Mapping the Presence and Conservation Status of Asbestos Containing Materials

Mapping of the presence and the conservation status of the coverings containing asbestos was performed in collaboration with AeroDron S.r.l. (Parma, Italy), in the framework of the project “Asbestos Free”^35^ within the eight municipalities of the “Unione dei Comuni della Bassa Reggiana” (Reggio Emilia province, Emilia Romagna region, Italy), that is, Boretto, Brescello, Gualtieri, Guastalla, Luzzara, Novellara, Poviglio, and Reggiolo.

In the first stage, aerial multispectral images (resolution of 300 ÷ 30 cm/pixel) were superimposed on cadastral maps to identify the coverings possessing spectral characteristics compatible with ACMs.

To refine the collected information, more defined images (resolution of 5 cm/pixel), were obtained by means of low-altitude drone surveys performed on those coverings presenting a nonuniform distribution of their spectral signature. After this refining stage, a first cluster including those coverings characterized by the maximum probability of being constituted of ACMs, was obtained together with a second cluster for which only on-site sampling and analyses can be used to certainly determine the presence of ACMs.

All the identified coverings were then georeferenced, linked to the relevant cadastral registry and to their size. A sequential alphanumeric ID was then assigned to each covering and stored in a database, including also their conservation status classification. Indeed, the data collected with the drone surveys were used to organize a conservation status classification of the coverings. The interpretation of the drone images allowed identifying flaking, cracks and further damages of the coverings. The assessment of the conservation status depended on how clear the degradation was and on the relative percentage of deteriorated covering with respect to the total surface. In this way, a risk map for the priority of intervention was created. By defining the damage probability (P) and the damage seriousness (S), both in a 1–3 absolute values range, the risk *R* was calculated as the product P·S and represented on a matrix with S reported on the *X*-axis and P reported on the *y*-axis. Priority of interventions was then scheduled according to the *R* value of the coverings. Coverings characterized by *R* ≥ 6 required immediate interventions, those with 3 ≤ *R* ≤ 4 required compelling interventions, while for those with *R* < 3 the interventions were programmed on a medium-term basis.

### Encapsulation and Removal of ACMs

According to the defined priorities of intervention, removal of ACMs was performed by Sabar Servizi S.r.l. (Reggio Emilia, Italy), authorized by the Italian register of the environmental managing institutions.^36^ The surfaces of ACMs were treated with a red-colored encapsulating agent (i.e., CEMBLOK BASE Performance, Venber-Geo Hydrica s.r.l, Verona, Italy) consisting in a water emulsion of artificial resins and additives, in compliance with the Italian legislation and with the Italian Ministerial Decree for penetrating encapsulating agents of type “D”.^37^ Therefore, once safely encapsulated the only possible asbestos fibers emissions were related to the necessary subsequent cutting operations for removing montage bolts.

Approximately 0.3 kg/m^2^ of this encapsulating agent were applied and left to dry for ca. 3 h. The operators were provided with the necessary individual protection devices. Removal of the as-encapsulated ACMs was performed both manually and by using portable battery-operating low-speed devices. According to the Italian legislation^38^ the as-obtained ACW was placed in double sealed waterproof polyethylene bags, of at least 0.15 mm thickness each, in order to avoid any possible asbestos fibers emissions during the following transport to the temporary storage plant.

The dust eventually present in the gutters of the buildings was removed by a wet method, consisting in the removal of the drain, wetting of the material with water, removal with shovel and final storage in sealed bags. All of the working areas and the relative access areas (e.g., roofs, floors, grounds beneath the covering, balconies, terraces, and stairs) were thoroughly cleaned by removing scraps and vacuuming the surfaces.

The waste materials resulting from all the cleaning operations together with the used personal protective equipment were put in the double-sealed bags, completely similar to those previously described for ACW. Packages not larger than *ca*. 1 m^3^ were prepared, to allow an easy transportation, and labeled in accordance with the Italian legislation. They were subsequently loaded on trucks and sent to a preliminary storage plant, to optimize transportation to their next end of life scenario.

The wastes received by the storage plant were classified according to the Italian legislation with the following codes: CER 170605* (i.e., construction materials containing asbestos), CER 170601* (i.e., insulating materials containing asbestos), CER 170603* (i.e., insulating materials containing or composed by other harmful substances) and CER 150202* (i.e., absorbents, filtering materials, rags, and protective clothing which are contaminated by harmful substances).

### First End of Life Scenario Considered: Thermal Inertisation Treatment Employing an Industrial Continuous Plant

The innovative end of life scenario investigated in this work for the stored ACW sealed packages consisted in their thermal treatment, prolonged for >24h, in a tunnel kiln at the temperature of 1200 °C, to obtain an asbestos-free product named KRY·AS, containing newly formed clinker phases.^33,39^

The incoming ACWs were scanned with X-rays in order to verify the presence of unwanted components. Trucks were then sent to the unloading area in the warehouse. In this indoor area, the packages were checked visually before being unloaded by the in-charge employees. Here, ACWs were then temporarily stored, in order to guarantee to the inertisation plant an autonomy of 2–3 months. This warehouse, with a capacity of 10 000–20 000 ton, was endowed with a continuous precautionary aspiration system, in order to avoid the spreading of dust and particles in the environment as a consequence of accidental events.

The methane-powered kiln has an average productivity of ca. 200 tons/day and ca. 78 000 tons/year. It is covered for the whole length and height with firebricks and semirefractory bricks. The firing cycle was completed in 38 h. It comprised an isothermal treatment at 1200 °C for 20 h. In the prefiring area of the kiln (when the temperature was raised from room temperature up to 1200 °C) the packaging materials were burnt. In the cooling area, the temperature was decreased from 1200 °C to approximately 30–35 °C. The kiln operates 7 days a week on three different shifts of 8 h each. The flue gases emitted by the kiln are treated and purified by a specific system including an afterburner. The flue gases emitted by the preheating area chimney pass through a baghouse filter and subsequently through a three-HEPA filters array in order to abate the overall dust. A ventilation system allows the flue gases to pass the HEPA filtering system and to reach the afterburner to eliminate possible organic substances (during this stage the flue gases reach a temperature of ca. 850 °C). A DeNO_*x*_ system is used to abate NO_*x*_.

The used filters, since they might contain asbestos fibers, were threated in the same furnace during the regular inertisation process for ACW.

The tunnel-shaped kiln works in a counter flow manner and is kept depressurized, in order for the flue gases to move to the preheating area, where the flue gas treatment system is positioned. The cooling area comprises two warm air intake systems, one for high temperature flue gases (at ca. 400 °C) and one for lower temperature flue gases (at ca. 105 °C). The first air intake system is paired to a Rankine-cycle operating turbine (cogeneration system) for the production of 250 kW of electricity and hot water. The electricity produced allows the whole plant to self-sustain, while the hot water obtained is directed into a district heating system for some suitable residential areas within the municipality where the plant is located.

The exiting KRY·AS inert material, whose average composition is reported and compared to the one of the incoming ACW in Table S1 of the Supporting Information (SI) (as determined by X-ray fluorescence spectroscopy, X-ray powder diffraction, and Rietveld method in previous works^40,41^) was subjected to a magnetic separation of ferrous materials (accounting for ca. 1% of the total entering material) and, if needed, to grinding operations to obtain the desired particle size distribution, according to the use thought for the as obtained secondary raw material. The as processed inert material was then sent to a temporary storage area, located indoor in a building close to the inertisation kiln. The storage area was equipped with water nebulizers to minimize dust emissions during loading/unloading operations.

### Second End of Life Scenario Considered: Disposal of ACW in Landfill for Hazardous Waste

The second end of life scenario considered was the disposal of asbestos containing waste in a landfill for hazardous waste, located in the province of Reggio Emilia, Italy, according to what established by the actual Italian legislation. As previously mentioned, after being encapsulated, removed and packaged, ACWs were transported to a storage plant, in order to optimize the transport to the landfill.

Once at the landfill, the packages of ACW were deposited in the dedicated lots, where a waterproofing system composed of different layers of clay, high density polyethylene (HDPE), polypropylene (PP) and sand was added for the final covering of the landfill.

## Life Cycle Assessment (LCA)

The LCA methodology was applied, according to the ISO 14040^42^ and 14044:^43^ its constituting phases are detailed hereafter.

### Goal and Scope Definition

#### Goal Definition

The goal of this study was to quantitatively assess the potential human toxicity and freshwater ecotoxicity impacts associated with the whole life cycle of the management of asbestos containing waste, found in the eight Italian municipalities. Two different treatment scenarios were considered, to identify the more environmentally sustainable (limitedly to the above-mentioned toxicological issues) management alternative.

The two different scenarios considered were (i) the thermal inertisation treatment of ACW employing an industrial continuous furnace, as recently patented by some of the present authors,^33^ leading to an inert material considered a secondary raw material for the production of porcelain stoneware slabs,^34^ and (ii) what is actually provided by Italian legislation, that is, ACW disposal in a controlled landfill for hazardous waste.

The nonmanagement scenario was also considered and compared with the previous ones, in order to highlight the necessity and urgency of intervention.

### System, Functional Unit, and Function of the System

The system object of the study is the management of ACMs in eight Italian municipalities. The functional unit selected is the amount of ACW that was collected and treated in the eight municipalities considered during a period of three months (i.e., 150 ton).

The function of the system is to contribute solving environmental and human health issues related to the presence of significant amounts of ACMs in the coverings of private and public buildings.

#### System Boundaries

**Figure 1:**
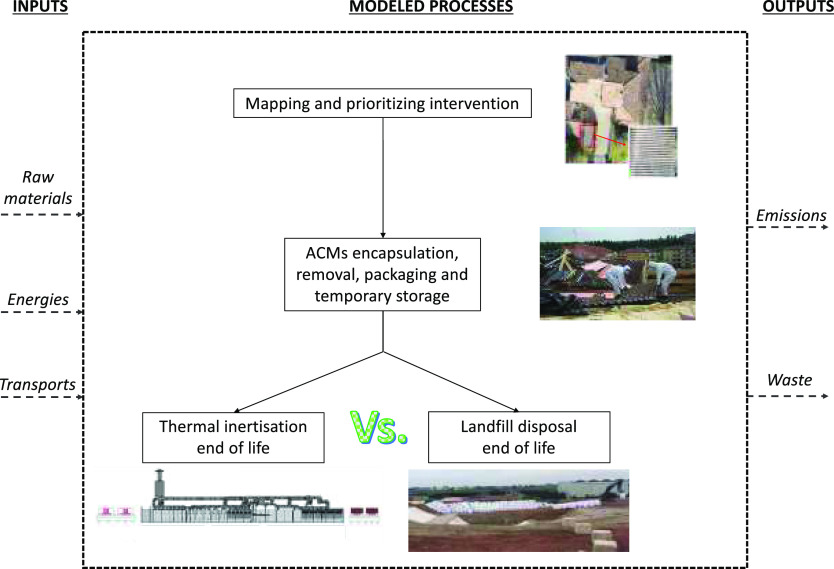
Flowchart showing the system boundaries considered in the LCA of the ACMs management.

The boundaries of the system investigated (i.e., the management of ACMs) include mapping of the presence and conservation status of ACMs in the eight Italian municipalities, their encapsulation and removal, together with two different scenarios for the treatment of the as obtained ACW, i.e. their thermal inertisation and their disposal in a landfill for hazardous waste. All the energies involved, the transport contributions, together with the emission into air, the local and the indoor emissions were considered as well. The system boundaries are summarized in Figure 1, and more extensively detailed in SI Figures S1–S4.

### Life Cycle Inventory (LCI) and Life Cycle Impact Assessment (LCIA)

Most of the data employed for the LCI phase were primary data, thus collected during the mapping and the removal activities performed. The inventory was completed with secondary data from the Ecoinvent database (EID, version 3.6),^44^ mainly to model the background processes (i.e., land use, materials production, fuel and electricity production, and materials transport). The EID processes employed were those characterized by an allocation at point of substitution (i.e., APOS processes), thus similar allocation of the impact between the product and the valuable coproducts was applied in the whole study also to those ad-hoc built processes, in order to make possible a fair comparison among the different management scenarios.

Primary data for the end-of-life scenario consisting in the thermal inertisation of ACW were derived from the recent patent by some of the present authors.^33^

Concerning the storage plant for the collected ACW and the hazardous waste landfill, data were obtained from the authors’ database, thus referred to a previous LCA study.^45^ That study referred to a landfill located in Castel Maggiore (Bologna, Italy) managing both hazardous and nonhazardous wastes, among which ACW. The data related to the monitoring of the landfill lifespan (considering an operational period of 15 years and a postclosure period of 30 years), that is, leachate production, air pollutants, and odors emitted from the landfill and surface water surrounding the landfill site, were collected directly from technician interviews, except for long-term emissions in groundwater which were gathered from residual material landfill data set of Ecoinvent.^44^ Those data, originally referred to the total mass of waste disposed into that landfill, were allocated to the functional unit selected in this study (i.e., 150 ton of ACW).

The main contributions to the LCI for the different phases of the processes are reported in SI Tables S2–S5, where the sources of data used for the considered amounts are indicated together with those of the background processes considered.

The inventory was modeled in SimaPro 9.1.1.1,^46^ by following an attributional modeling, without applying any cutoff criteria. Due to the multifunctional character of the inertisation treatment, it was modeled applying a multioutput scenario, with the production of a coproduct made of inert material to be used in the production of porcelain stoneware slabs. Particularly, the thermal inertisation treatment of 150 ton of ACW allowed the obtainment of 112.5 ton of KRY·AS inert material. Although the use of KRY·AS in the production of porcelain stoneware slabs was demonstrated,^34^ the absence of dedicated regulations for these secondary raw materials makes them still lacking an own market, so that a mass allocation criterion was applied. Particularly, the human and ecotoxicity impacts calculated were allocated for the 57.14% to the thermal inertisation of 150 ton of ACW and for the 42.86% to the KRY·AS material obtained. However, its comparison with the landfill disposal scenario (the latter being a single output process) was also reported without discounting it with any damage percentages.

The human toxicity (for both carcinogenic and noncarcinogenic substances) and the freshwater ecotoxicity were calculated by applying the USEtox method.^29^ USEtox is a scientific consensus LCIA model, which has been developed since 2003 under the auspices of the United Nations Environment Programme–Society of Environmental Toxicology and Chemistry Life Cycle Initiative as a harmonized approach for characterizing human and freshwater toxicity in life cycle assessment and other comparative assessment frameworks.^47^ Therefore, USEtox model was adopted to identify the characterization factors for assessing the potential effects on human health caused by asbestos emissions in both indoor (occupational settings) and outdoor air environments.

USEtox defines the characterization factor (CF) of a substance as a quantitative representation of how hazardous that substance is or potentially impacts, in relation to the emission of a unit mass of a pollutant.^48^ For each substance its midpoint CF is calculated as reported in eq 1, considering the fate factor (FF), the exposure factor (XF) and the effect factor (EF) of the emitted substance.^49,50^ The end point CF can then be obtained by multiplying the as determined midpoint CF by the severity factor (SF) in order to obtain the damage assessment.^51,52^
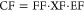
1

This study on purpose focuses on the human carcinogenicity related only to direct exposure by inhalation to asbestos fibers. Indeed, in addition to the fact that asbestos does not accumulate in the food chain and that neither biomagnification process occurs,^53^ to the best of authors’ knowledge the potential adverse effects associated with ingestion of asbestos fibers (e.g., gastrointestinal carcinogenicity) is still the object of debate with several contradictory results,^54,55^ so that the IARC still considers data on the risk of gastrointestinal cancer not conclusive.^56^

During the creation of a new substance in the USEtox model file worksheets, some substance-specific data, that is, physical–chemical properties, environmental degradation, and human toxicity, must be included.

Particularly, in this work some physical–chemical properties and degradation rates of carbon nanotubes proposed by Rodriguez-Garcia et al.^57^ were used and implemented in the USEtox worksheets in order to model the fate factor of asbestos fibers.

This choice can be scientifically justified by considering that some forms of carbon nanotubes (CNTs) were recognized since 2004^58^ as possessing physical similarities with respect to asbestos fibers, mainly in terms of the parameters dictating whether or not an inhaled fiber will be pathogenic. These latter parameters were recognized by the fiber pathogenicity paradigm (FPP),^59^ as being width, length, and biopersistence. Thus, FPP independence from composition (except when composition contributes to biopersistence) makes this paradigm embracing also CNTs. However, since different forms of CNTs exist, the FPP only pertains to high aspect ratio CNTs, thus possessing a fibrous shape.^60^

As concerning biopersistence, pristine carbon nanotubes were proven to be extremely durable by using in vitro assays,^61^ with multiwalled carbon nanotubes (MWCNTs) being significantly less amenable to degradation with respect to single-walled carbon nanotubes (SWCNTs). Due to the above-mentioned similarities, some physical and chemical properties of MWCNTs were used as proxies for fate and exposure parameters as well as for ecotoxicity of asbestos fibers in USEtox, as detailed in SI Table S6.

Moreover, human toxicity value ED50_inh, can_ (human equivalent lifetime dose that would cause a cancer probability of 50% after inhalation (kg·lifetime^–1^)) needs also to be inserted in the USEtox workbook to assess the effect factor. For carcinogenic effects, the ED50 has been estimated from the *carcinogenic*, *low-dose*, slope factorq* by the 1/q*-to-ED50 extrapolation factor, which is equal to 0.8^53^ as recommended by USEtox method. A slope factor q* equal to 2.2 × 10^2^ (mg/kg·day)^−1^ was here taken into account.^62^ Therefore, the ED50_inh, can_ resulted 6.5 × 10^–3^ [kg·lifetime^–1^], corresponding to an Effect Factor _inh, can_ of 7.69 × 10^1^ (cases·kg_intake_^–1^) that is similar for example to the one of the well-known carcinogen Benzo[a]pyrene (i.e., 7.30 × 10^1^ (cases·kg_intake_^–1^), according with the USEtox worksheet). The obtained midpoint CFs for asbestos fibers are reported in Table 1, for both indoor (used to mimick the occupational setting) and outdoor air compartments.

**Table 1:** Midpoint Characterization Factors for Asbestos Fibers Related to Human Toxicity, Carcinogenic Effects

indoor air (occupational setting)	outdoor air
(cases/kg_emitted_)	(cases/kg_emitted_)
1.07 × 10^–2^	1.63 × 10^–4^

In this way, the new substances named asbestos fibers and asbestos fibers indoor were characterized in the USEtox method, in order to assess also their contribution to the human toxicity impacts of the whole management scenarios investigated.

Particularly, the masses of asbestos fibers released in outdoor air and in an occupational setting will be multiplied by these CFs, thus obtaining their contributions (expressed in cases) to the human toxicity, cancer impact category (i.e., the only one that was considered affected by asbestos fibers).

## Results and Discussion

LCIA results were obtained by the USEtox evaluation method, in order to compare the impacts on human toxicity and freshwater ecotoxicity of the two different management scenarios for asbestos containing materials, that is, the one comprising the thermal inertisation treatment of the derived ACWs and the one involving their disposal in landfill for hazardous waste.

The possibility offered by the innovative thermal inertisation treatment applied to 150 ton of ACW to obtain 112.5 ton of KRY·AS secondary inert raw material usable in the production of porcelain stoneware slabs (thus exiting the boundaries of the system), allows to reduce the toxicity impact associated with this management solution for ACMs. Indeed, by considering the mass allocation criterion used to model the process, only the 57.14% of the whole environmental damage must be attributed to the management solution proposed.

**Figure 2:**
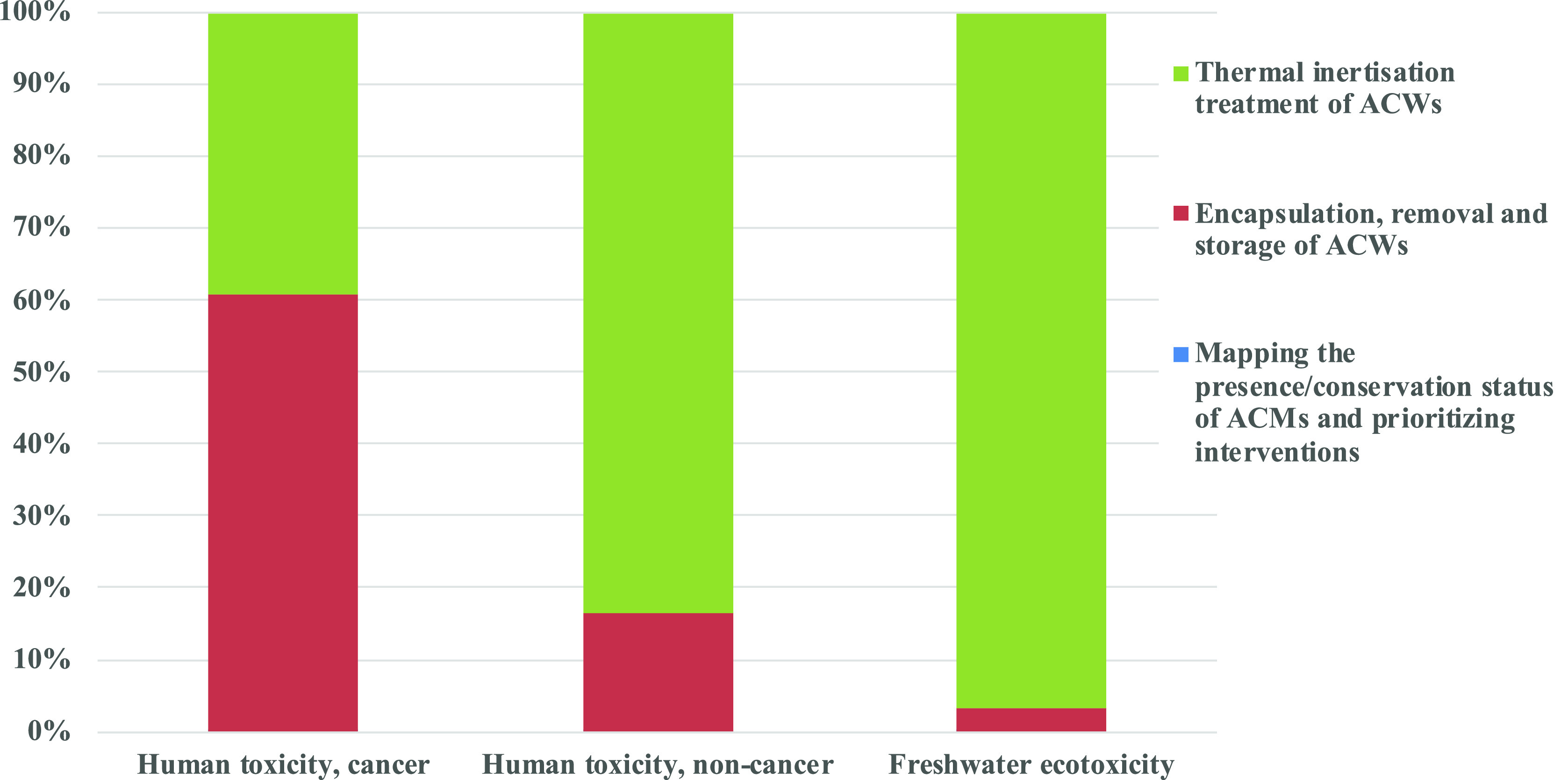
Evaluation by impact categories of the ACMs management solution comprising the innovative thermal inertisation end of life scenario of 150 ton of ACW.

The results reported in Figure 2 and quantitatively detailed in SI Table S7 show that the life cycle phase with the highest environmental load (limitedly to the impact categories considered by USEtox) is the innovative end of life scenario proposed in this study, i.e. the thermal inertisation treatment. It contributes for 96.9% (i.e., for 1.90 × 10^9^ PAF·m^3^·day) to the Freshwater ecotoxicity impact category, for 83.5% (i.e., for 3.74 × 10^–3^ cases) to the Human toxicity, noncancer one and for 39.1% (i.e., for 1.30 × 10^–3^ cases) to the Human toxicity, cancer one. The second for importance phase affecting the whole environmental load considered is the encapsulation and removal of ACMs. It is responsible for more than 60% of the human toxicity, cancer impact category (i.e., for 2.03 × 10^–3^ cases).

By focusing on human toxicity, cancer impact category, its whole damage is 3.33 × 10^–3^ cases and it is mainly (for 39.9%) due to Chromium VI in water that is generated by the thermal inertisation treatment, as a consequence of the background ecoinvent process used to model the end-of-life treatment of the materials retained by the filters (i.e., filter dust from Al electrolysis {CH}| treatment of filter dust from Al electrolysis, residual material landfill |APOS, U). Indeed, this waste treatment process consists in a residual material landfill for polluted, inorganic waste comprising base seal and leachate collection system, as well as the recultivation of the soil after its closure.

Second, the 32.6% and the 21.6% of the impact on the category human toxicity, cancer are due to asbestos fibers released in air and inhaled by the workers, respectively. They are both totally released during the encapsulation and removal phase. In detail, 6.727 kg is the portion of the total amount of asbestos fibers released in air compartment, attributable to the thermal inertisation treatment. The 1 wt % (i.e., 0.067 kg) of these asbestos fibers was assumed to be inhaled by the workers (considered to be emitted in indoor air compartment).

The fact that no asbestos fibers can be found in the gaseous emissions released during the thermal inertisation procedure itself was recently demonstrated by Tomassetti et al.,^63^ concurrently with the confirmation of their absence in the solid residue. Particularly, SI Appendix A summarizes the results of the analysis performed on the collected emissions of particulate, supplied also with meaningful micrographs (SI Figures S5–S12). As described in SI Appendix A, the only fibers detected resulted exotic ceramic fibers released from the refractory medium used to better isolate the furnace. Their potential hazard (mainly in terms of the FPP) was, however, not characterized due to the not quantifiable rarity of the phenomenon, as well as the fact that those fibers were indeed retained by the filters of the thermal inertisation plant, together with the intrinsic possibility to investigate the use of different refractory materials.

Concerning the human toxicity, noncancer, impact category, its whole damage is 4.48 × 10^–3^ cases. Mercury in air and arsenic in water generate the main environmental loads (34.6% and 24.9% respectively) and they are mostly associated with the process thermal inertisation treatment (for 90.7% and for 79.2% respectively). Mercury is released during the production of sodium hydroxide (the EID process considered was: Sodium hydroxide, without water, in 50% solution state {RER}| chlor-alkali electrolysis, mercury cell) that is necessary for the exhaustion of CO_2_ produced in the afterburner, whereas arsenic emission is associated with the EID waste treatment process considered to model the end of life of the materials retained by the filters of the thermal inertisation plant (i.e., filter dust from Al electrolysis (waste treatment) {CH}| treatment of filter dust from Al electrolysis, residual material landfill). To the same EID background process is also due the emission of aluminum in water, that determines the main environmental load (97.5%) of the freshwater ecotoxicity impact category.

Although the thermal inertisation treatment of ACWs, represents the most impacting phase of the whole management of ACMs, by comparing it with what actually provided by Italian legislation, that is, disposal of ACWs in a controlled landfill for hazardous wastes, the results reported in Table 2 are obtained, from which the significantly higher impact of the second end of life scenario results evident, in terms of human toxicity and freshwater ecotoxicity. For reasons of completeness and to furnish a more fair comparison, Table 2 also reports the impacts of the management scenario comprising the thermal inertisation treatment, without considering the mass-based allocation (i.e., the whole damage is now to be attributed to the function of the system).

**Table 2:** Detailed Quantitative LCIA Comparison for the Two ACMs Management Scenarios Considered, Differing in the Sole End of Life Phase Considered for the 150 Ton of ACW^a^

impact category	unit	residual material landfill	thermal inertisation treatment (allocated)	thermal inertisation treatment (not allocated)
human toxicity, cancer	cases	2.18 × 10^–1^	3.33 × 10^–3^	5.83 × 10^–3^
human toxicity, noncancer	cases	1.34 × 10^–2^	4.48 × 10^–3^	7.85 × 10^–3^
freshwater ecotoxicity	PAF·m^3^·day	1.58 × 10^10^	1.96 × 10^9^	3.43 × 10^9^

a For the management scenario comprising the thermal inertisation treatment both mass-based allocated results and results not allocated at all, are reported.

The management scenario comprising the ACWs disposal in a landfill for hazardous waste, produces a higher environmental damage with respect to all the impact categories of USEtox.

In detail, the emissions into groundwater that determine the higher impacts are (i) chromium VI contributing for 98.5% to human toxicity, cancer impact category, (ii) vanadium contributing for 84.1% to human toxicity, noncancer impact category and (iii) aluminum, contributing for 99.3% to freshwater ecotoxicity category. These metal emissions are all associated with the Ecoinvent process employed to model the long-term emissions associated with the landfill for hazardous waste, as detailed in SI Table S5.

As inferable from the data of Table 2, the higher discrepancy between the two end of life scenarios is the one referred to Human toxicity, cancer impact category. As discussed above, this is mainly due to the long-term emissions in groundwater associated with the residual material landfill. Moreover, a further contribution is due to the asbestos fibers released in air, since this is the only USEtox impact category possessing an additional asbestos-related characterization factor. Table 3 summarizes the amounts of asbestos fibers released during each phase of the management scenarios considered and the corresponding impacts to the Human toxicity, cancer impact category. As reported in Table 3, in the management scenario comprising the residual material landfill end of life, the total amounts of 11.65 kg of asbestos fibers released in air and 0.12 kg inhaled by the workers (both associated with encapsulation/removal phase, see also SI Table S3) are now completely to be attributed to the disposal of ACWs in the landfill for hazardous wastes, since no useful coproducts are generated by this traditional end of life scenario.

**Table 3:** Summary of the Amounts of Asbestos Fibers Released during Each Management Scenario and the Corresponding Impacts to the Human Toxicity, Cancer Impact Category

management scenario comprising the final residual material landfill					management scenario comprising the thermal inertisation treatment							
					with mass-based allocation				without allocation			
phase	asbestos fibers emitted (kg)		impact of the emitted fibers on Human toxicity, cancer (cases)		asbestos fibers emitted (kg)		impact of the emitted fibers on Human toxicity, cancer (cases)		asbestos fibers emitted (kg)		impact of the emitted fibers on Human toxicity, cancer (cases)	
	outdoor	indoor	outdoor	indoor	outdoor	indoor	outdoor	indoor	outdoor	indoor	outdoor	indoor
mapping and prioritizing interventions	/	/	/	/	/	/	/	/	/	/	/	/
encapsulation removal and storage	11.65	0.12	1.89 × 10^–3^	1.259 × 10^–3^	11.65	0.12	1.085 × 10^–3^	7.193 × 10^–4^	11.65	0.12	1.898 × 10^–3^	1.259 × 10^–3^
end of life	3.98 × 10^–3^	/	0.01 × 10^-4^	/	/	/	/	/	/	/	/	/
total	11.77		3.15 × 10^–3^		11.77		1.80 × 10^–3^		11.77		3.157 × 10^–3^	

Moreover, a further amount of 3.98 g of asbestos fibers is released in air during the disposal of ACWs in the controlled landfill for hazardous wastes (see also SI Table S5).

Therefore, at least in the particular case of ACMs management in the eight municipalities object of the present study, the proposed thermal inertisation end of life must be considered a valuable and more environmentally sustainable alternative to traditional disposal in dedicated landfills, limitedly to toxicity related burdens. It is however to be highlighted that the main responsible for the higher impact of the residual material landfill disposal scenario is indeed the process used to model the long-term emissions in groundwater, associated with the end of life of the landfill itself. Indeed, as inferable from the data reported in Table 3, without applying any allocation criteria to the thermal inertisation treatment comprising scenario, the contribution of the asbestos fibers to the human toxicity, cancer impact category is only slightly lower with respect to the landfill disposal comprising scenario.

However, independently by the above-mentioned allocation-criteria based considerations, the necessity and urgency of intervention, from an asbestos fiber related human health perspective, can be highlighted by considering the implications related to the not-removal at all of ACMs at a National scale. Indeed, in this latter case the asbestos fibers would be completely released over a given time frame, due to the unavoidable degradation of the coverings made of ACMs, occurring as a consequence of their natural degradation and/or catastrophic events.

**Figure 3:**
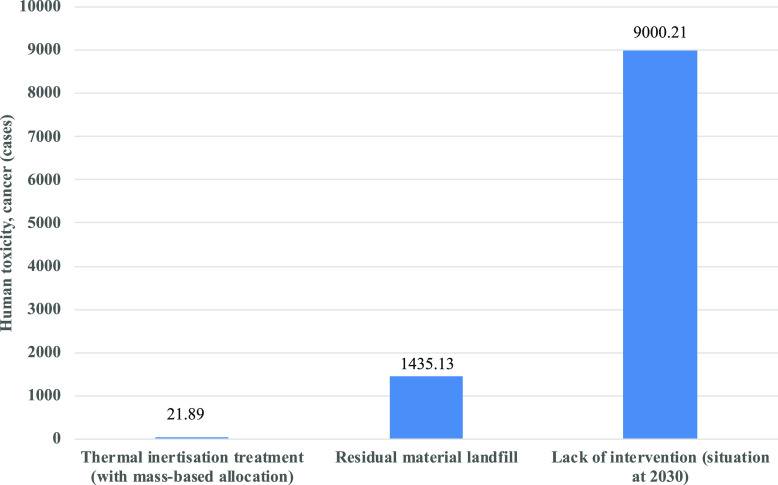
LCIA comparison (limited to human toxicity, cancer impact category) between the two management scenarios considered with the not removal at all of ACMs. The calculation was performed for the same functional unit, that is, the 58 millions of square meters of ACMs still present in Italy, corresponding to 986 000 ton of ACMs with an average asbestos fibers content of 10 wt %. For the lack of any kind of intervention, the hypothesized asbestos fibers emission scenario at the year 2030 is reported (details can be found in SI Appendix B).

By considering the data from a recent survey, approximately 58 millions of square meters of ACMs are still present in Italian public and private buildings as well as industrial sites.^14^ Therefore, by comparing the two studied management scenarios (i.e., thermal inertisation treatment and landfill disposal) with the lack of removal of this amount of ACMs needing intervention, the hypothesized 2030 scenario in terms of human health, cancer impact category, is reported in Figure 3, underlying a tremendously higher number of potential cancer cases associated with the absence of any kind of intervention. The considerations and the necessary assumptions made to perform this evaluation can be found in the SI Appendix B.

Overall, the main implications of the present study are necessarily related to the proposal of a characterization factor for asbestos fibers released both in outdoor air and indoor air compartments, that allow to account for this carcinogenic substance in the quantitative assessment of human health impact. Although, this characterization factor was calculated by employing both asbestos related data as well as data related to MWCNTs (due to their well-documented similarities), its effect factor resulted similar to the one of further well-known carcinogens, so that it can be considered a first attempt toward a desirable always more and more rigorous and reliable characterization of this harmful substance.

Its implementation in USEtox 2.0 impact assessment method allowed to quantitatively compare two management scenarios for ACMs differing only in the end-of-life phase for ACWs. Particularly, a scenario comprising the thermal inertisation treatment of ACWs was compared with the one comprising the landfill disposal of ACWs. The human and ecotoxicological impacts of the first scenario were reduced by 42.48%, since it allows the production of an inert secondary raw material. Despite, the results were significantly affected by the here applied mass-based allocation criterion, it needs to be emphasized that no reliable economic allocation could be applied, due to the lack of dedicated regulations for the use of such KRY·AS material in the construction sector. Moreover, the selected mass-based allocation led to an almost equal distribution of the impacts between the function of the system (i.e., the thermal inertisation treatment) and the obtained coproduct (i.e., KRY·AS inert material), that for a recycling process (as the present one can be easily considered) is a reasonable choice, also in a circular economy oriented development philosophy.

However, when the allocation criterion was not applied, the scenario comprising the landfill disposal of ACWs still resulted more impacting with respect to the thermal inertisation based alternative.

Although the study was performed mostly by employing primary data, the uncertainties related to the unavoidable use of background processes, require surely to be carefully evaluated.

Particularly, the main differences observed between the human carcinogenicity impacts of the two management scenarios assessed in the present work, resulted related to the long-term emissions in groundwater associated with the landfill disposal scenario, rather than to asbestos fibers.

On the opposite, the impact of asbestos fibers to human toxicity, cancer impact category significanly contributed to highlight the necessity and urgency of removal intervention, rather than the lack of any intervention at all, irrespective of both the management scenario considered and the allocation criteria applied. This is due to the unavoidable complete release of asbestos fibers over a given time frame, as a consequence of the natural degradation of ACMs-made coverings and/or the occurrence of catastrophic events.

## Supporting Information Available

The average phase composition for incoming ACW and the thermally transformed KRY·AS inert material is reported in Table S1. Tables S2–S5 report the main contributions to the LCI phase of this study. The main properties and parameters used to model asbestos fibers in USEtox are reported in Table S6. The detailed LCIA results for the ACMs management solution comprising the thermal inertisation treatment end of life is reported in Table S7. Figures S1–S4 depict the details of the boundaries of the systems investigated. Appendix A summarize the analyses performed on the particulate emissions during the thermal inertisation treatment, presenting typical SEM micrographs as Figures S5–S12. Appendix B summarize the considerations and assumptions made for the calculation of the asbestos fibers released as a consequence of the lack of any removal intervention (PDF)
