# Supplementary material for: Management of Asbestos Containing Materials: A Detailed LCA Comparison of Different Scenarios Comprising First Time Asbestos Characterization Factor Proposal
Source: Environ Sci Technol. 2021 Sep 1;55(18):12672–82. doi: 10.1021/acs.est.1c02410 (PMC8459455; doi:10.1021/acs.est.1c02410)
Supplement: Supplementary file 1 — es1c02410_si_001.pdf [file es1c02410_si_001.pdf]

SUPPORTING INFORMATION

Management of asbestos containing materials: a detailed LCA comparison of different scenarios comprising first time asbestos characterization factor proposal

Martina Pini[a,b], Simone Scarpellini[a], Roberto Rosa[a,b,*], Paolo Neri[a], Alessandro F. Gualtieri[c], Anna Maria Ferrari[a,b]

[a]Department of Sciences and Methods for Engineering, University of Modena and Reggio Emilia, Via G. Amendola 2, 42122, Reggio Emilia, Italy

[b]Interdepartmental Center En&Tech, University of Modena and Reggio Emilia, Via G. Amendola, 2, 42122 Reggio Emilia, Italy

[c]Department of Chemical and Geological Sciences, University of Modena and Reggio Emilia, Via G. Campi 103, 41125, Modena, Italy

*corresponding author: Tel.: +390522523558; E-mail: roberto.rosa@unimore.it

Number of pages: 28

Number of Tables: 7

Number of Figures: 12

Table S1. Average phase composition for incoming ACW and thermally transformed product at 1200 °C (KRY·AS), as determined by X-ray fluorescence (XRF), X-ray powder diffraction and the Rietveld method.

Phase	Raw ACW (wt%)	KRY·AS (wt%)	Phase	Raw ACW (wt%)	KRY·AS (wt%)
Chrysotile	1.4-13.3	-	Mayenite	-	0-7.3
Amphibole asbestos	0-6.5	-	Lime	-	0-6.9
Calcium carbonates	0-68.8	-	Periclase	-	0-7.7
Quartz	0-1.9	0-9.4	Calcium sulphates	0-5.2	-
Cristobalite	-	0-0.6	Ternesite	-	0-11.3
Feldspars	0-2.9	-	Portlandite	0-10.9	0-1.5
Wollastonite	0-0.9	0-3.8	Yeelimite	-	0-8.3
Pseudo-wollastonite	-	0-9.9	Jarosite	0-1.8	-
Mica+illite	0-2.2	-	Ettringite	0-2.5	-
Belite-β	0-7.1	-	hydrogarnets	0-2.0	-
Alite-M_3/C3S*	0-3.6	0-2.0	AFmc***	0-2.0	-
Larnite	-	4.8-71.0	Diopside	-	0-7.2
Ferrite/C4AF**	0-2.5	0-8.8	Forsterite	-	0-2.9
Aluminate	0-1.3	-	Enstatite	-	0-3.4
Rankinite	-	0-11.0	Magnetite	-	0-1.3
Akermanite	-	0-58.0	Hematite	-	0-0.7
Bredigite	-	0-31.6	Maghemite	-	0-3.2
Merwinite	-	0-19.2	Amorphous phase	9.5-66.6	3.4-19.2
Monticellite	-	0-1.6			

*C3S = tricalcium silicate, Ca_3SiO_5; **C4AF = Tetracalcium alumino ferrite, $4{\cdot}CaO{\cdot}Al_2O_3{\cdot}Fe_2O_3$; ***AFmc = calcium monocarboaluminate hydrate.

Table S2. Main contributions to the Life Cycle Inventory for 150 ton of ACMs mapping and prioritizing intervention activities.

Description			Amount	Process data source
Input	Equipment	Aerial flight	1.42×10^{-4} h	EID[1]
		Camera	2.21×10^{-6} kg	EID[2]
		Laptop	2.53×10^{-6} p	EID[3]
		Drone	3.18×10^{-3} kg	EID[4]
		Li-ions battery	7.86×10^{-2} g	EID[5]
	Energy	Electric energy	0.70 kWh	EID[6]
	Transport	Equipment transport	1.42×10^{-2} tkm	EID[7,8]
Output	End of life	Waste treatment (camera end of life)	2.21×10^{-6} kg	EID[9]
	End of life	Recycling (airplane end of life)	1.42×10^{-1} kg	EID[10]

[1]The EID process used was: Transport, helicopter {GLO}| processing.
[2]The EID process used was: Electronic component, active, unspecified {GLO}| production.
[3]The EID process used was: Computer, laptop {GLO}| production.
[4]The EID process used was: Polypropylene, granulate {RER}| production.
[5]The EID process used was: Battery, Li-ion, rechargeable, prismatic {GLO}| production.
[6]The EID process used was: Electricity, low voltage {IT}| electricity voltage transformation from medium to low voltage.
[7]The EID process used was: Transport, freight, lorry 16-32 metric ton, EURO6 {RoW}| transport, freight, lorry 16-32 metric ton, EURO6.
[8]An average distance of 100 km was considered for the road freight transport by lorry of equipment.
[9]The EID process used was: Used industrial electronic device {GLO}| treatment of, mechanical treatment.
[10]A 50:50 wt% mix of the following two EID processes was used: Aluminium, cast alloy {RoW}| treatment of aluminium scrap, new, at refiner, and Steel, low-alloyed {RER}| steel production, electric, low-alloyed.

Table S3. Main contributions to the Life Cycle Inventory for 150 ton of ACMs safe encapsulation, removal, packaging and preliminary storage of ACWs before their end of life scenario.

Description			Amount	Process data source
Input	Materials	Encapsulating agent	1.42 ton	EID[1]
		Packaging	1.08 ton	EID[2]
	Equipment	Crane truck	$1.40x10^{-4}$ p	EID[3]
		Warehouse	$3.78x10^{-5}$ p	Modeled from EID sub processes
		LGV (Laser Guided Vehicle)	$1.08x10^{-4}$ p	Modeled from EID sub processes
	Processes	Packaging manufacturing	1.08 ton	EID[4]
		Operation of commercial generator	9.46 h	EID[5]
	Land occupation/transformation	Land occupation	2.25 m^2·year	EID[6]
		Land transformation	$4.51x10^{-2}$ m^2	EID[7]
		Land transformation	$4.51x10^{-2}$ m^2	EID[8]
	Energy	Electric energy	294.12 kWh	EID[9]
	Transport	Equipment transport	0.10 tkm	EID[10,11]
		Materials transport	3424.70 tkm	EID[12]
Outputs	End of life	Steel bolts recycling	0.85 kg	EID[13]
	Emissions	Asbestos fibers	11.65 kg[14]	Simapro airborne emission substance list
		Asbestos fibers (indoor)	0.12 kg[15]	Simapro airborne emission substance list
		Particulates, <2.5 µm	104.82 kg[16]	Simapro airborne emission substance list
		Particulates, <2.5 µm (indoor)	1.06 kg[17]	Simapro airborne emission substance list

[1]The encapsulating agent employed (i.e. CEMBLOK BASE® Performance, Venber®-Geo Hydrica s.r.l., Verona, Italy) complies with the Italian Ministerial Decree for penetrating encapsulating agents of type “D” [1], therefore once safely encapsulated the only possible asbestos fibers emissions are related to the necessary subsequent cutting operations for removing montage bolts. The EID process used was: Epoxy resin insulator, SiO_2 {RoW}| production.
[2]According to the Italian legislation [2] the obtained ACW was placed in double sealed waterproof polyethylene bags, of at least 0.15 mm thickness each, in order to avoid any possible asbestos fibers emissions during the following transport to the temporary storage plant. The EID process used was: Polyethylene, linear, low density, granulate {RER}| production.
[3]The EID process used was: Mobile cable yarder, truck-mounted, incl. processor {GLO}| cable yarder production, truck-mounted.
[4]The EID process used was: Blow moulding {RoW}| production.
[5]The EID process used was: Machine operation, diesel, >=74.57 kW, generators {GLO}| machine operation, diesel, >= 74.57 kW, generators.
[6]The EID process used was: Occupation, industrial area, built up.
[7]The EID process used was: Transformation to industrial area, built up.
[8]The EID process used was: Transformation from construction site.
[9]The EID process used was: Electricity, low voltage {IT}| electricity voltage transformation from medium to low voltage.
[10]An average distance of 100 km was considered for the road freight transport by lorry of equipment.
[11]The EID process used was: Transport, freight, lorry 16-32 metric ton, EURO6 {RoW}| transport, freight, lorry 16-32 metric ton, EURO6.
[12]The EID processes used were: Transport, freight, lorry 3.5-7.5 metric ton, EURO6 {RER}| transport, freight, lorry 3.5-7.5 metric ton, EURO6, and Transport, freight, lorry >32 metric ton, EURO6 {RER}| transport, freight, lorry >32 metric ton, EURO6.
[13]The EID process used was: Steel, low-alloyed {RER}| steel production, electric, low-alloyed.
[14]For the calculation of asbestos emissions, first of all it has been considered that on average ACMs contain 10 wt% of asbestos fibers, in accordance with literature [3]. Moreover, this value was deliberately considered slightly higher (for a precautionary approach) with respect to the average wt% of Chrysotile and Amphibole asbestos present in the experimentally characterized ACWs, as previously detailed in Table S1. Secondly, it has been considered that 5 g of ACMs (containing 10 wt% of asbestos fibers) are removed during each slab cut performed to remove montage bolts (this operation representing the only occasion for asbestos fibers emission). This value was furnished by technicians of Sabar Servizi S.r.l. (Reggio Emilia, Italy), i.e. the company in charge of the removal of ACMs [4].
[15]It has been estimated that 1 wt% of removed asbestos fibers remain in the indoor environment, directly affecting the operators.
[16]It has been estimated that the remaining 90 wt% of ACMs removed during each slab cut is composed of Particulates, <2.5 μm.
[17]It has been estimated that 1 wt% of the released Particulates (<2.5 μm) remains in the indoor environment, directly affecting the operators.

Table S4. Main contributions to the Life Cycle Inventory for the first end of life scenario considered for the 150 ton of ACWs, i.e. their thermal inertisation treatment employing an industrial continuous plant.[1]

Description			Amount	Process data source
Outputs: avoided products	Energy	Electric energy	4166.67 kWh	EID[2]
		Heat	40636.20 kWh	EID[3]
Inputs	Energy	Electric energy	4166.67 kWh	EID[2]
		Heat	345481.13 MJ	EID[4]
		Heat (afterburner)	2242.97 MJ	EID[5]
		Electric energy (afterburner)	1800.26 kWh	EID[6]
	Equipment	Chemical hood	$2.09x10^{-4}$ p	Modeled from EID sub processes
		Baghouse filter	$1.39x10^{-2}$ p	Modeled from EID sub processes
		Factory	$2.96x10^{-4}$ p	EID[7]
		Chemical hood (afterburner)	$8.47x10^{-2}$ p	Modeled from EID sub processes
		CO_2 scrubber (afterburner)	$4.23x10^{-2}$ p	Modeled from EID sub processes
		NO_x scrubber (afterburner)	$4.23x10^{-2}$ p	Modeled from EID sub processes
	Processes	Grinding and iron removal	49.34 ton	Modeled from EID sub processes
	Materials	NaOH (for the CO_2 scrubber)	$2.67x10^{3}$ kg	EID[8]
		NH_3 (for the NO_x scrubber)	9.29 kg	EID[9]
	Transport	Equipment transport	49.16 kgkm	EID[10,11]
		Materials transport	$1.20x10^{6}$ kgkm	EID[10,11]

Outputs	Emissions[12]	Hydrogen cyanide	0.22 g	Simapro airborne emission substance list
		CO_2, fossil	880.95 g	Simapro airborne emission substance list
		Ammonia	2786.46 g	Simapro airborne emission substance list
		Nitrogen oxides	6228.55 g	Simapro airborne emission substance list
		Nitrogen, atmospheric	3.13×10^5 g	Simapro airborne emission substance list
	End of life	Waste treatment (materials retained by the filters)	7.07×10^6 g	EID[13]

[1] The original primary data were referred to the ton of ACW treated by the plant in 24 h (i.e. 216 ton). Thus, a time-based allocation was employed considering a single inertization cycle lasting 38 h (i.e. 216ton/24h*38h=342 ton). Concerning the equipment, those processes were allocated considering their lifetime (i.e. 1p/lifetime of the equipment (h)*38h). Finally, since the functional unit of the study is 150 ton, a mass-based allocation was applied (i.e. the original amounts were further multiplied by a factor equal to 1/342*150).

[2]The EID process used was: Electricity, high voltage {IT}| heat and power co-generation, natural gas, combined cycle power plant, 400 MW electrical.

[3]The EID process used was: Heat, district or industrial, natural gas {IT}| heat and power co-generation, natural gas, combined cycle power plant, 400 MW electrical.

[4]The EID process used was: Heat, district or industrial, natural gas {RoW}| heat production, natural gas at industrial furnace >100 kW.

[5]The EID process used was: Natural gas, burned in industrial furnace >100kW.

[6]The EID process used was: Electricity, low voltage {IT}| market for.

[7]The EID process used was: Composite working factory/RER/I.

[8]The EID process used was: Sodium hydroxide, 50% in H_2O, production mix, at plant/RER.

[9]The EID process used was: Ammonia, liquid {RER}| market for.

[10]The EID process used was: Transport, lorry 20-28 t, fleet average/CH.

[11]An average distance of 100 km was considered for the road freight transport by lorry of both equipment and materials.

[12]No emissions of asbestos fibers were considered during this end of life scenario, since they were experimentally determined as absent in the work by Tomassetti et al. [5], both in the gaseous emissions released during the thermal inertisation procedure as well as in the solid residue. Particularly, Appendix A reported at the end of SI section summarizes the results of the analysis performed on the collected emissions of particulate, supplied also with meaningful micrographs (Figures S1-S8).

[13]The EID process used was: Filter dust from Al electrolysis (waste treatment) {CH}| treatment of filter dust from Al electrolysis, residual material landfill.

Table S5. Main contributions to the Life Cycle Inventory for the second end of life scenario considered for the 150 ton of ACWs, i.e. their disposal in a controlled landfill for hazardous waste.

Description			Amount	Process data source
Outputs, avoided products	Materials	Waste from incinerators used for the final covering[1]	0.18 ton	EID[2]
Inputs	Land occupation/transformation	Land occupation	30.39 m^2·year	EID[3]
		Land occupation	91.18 m^2·year	EID[4]
		Land occupation	5.47 m^2·year	EID[5]
		Land transformation	6.08 m^2·year	EID[6]
		Land transformation	6.08 m^2·year	EID[7]
		Land transformation	0.11 m^2·year	EID[8]
		Land transformation	6.08 m^2·year	EID[9]
	Materials for vapors treatment	NaClO	1.12×10^{-3} ton	EID[10]
		H_2SO_4	4.02×10^{-3} ton	EID[11]
		$Na_5P_3O_{10}$	9.55×10^{-5} ton	EID[12]
		$(NH_4)_2CO_3$	3.3×10^{-4} ton	EID[13]
	Materials for final covering of the landfill	Sand	1.37 ton	EID[14]
		Clay	3.83 ton	EID[15]
		Bentonite	0.05 ton	EID[16]
		Polypropylene	0.02 ton	EID[17]
		HDPE	5.84×10^{-3} ton	EID[18]
	Processes	Spinning of polypropylene	0.02 ton	EID[19]
		Weaving of polypropylene	0.02 ton	EID[20]

	Transport	Materials transport	527.84 t·km	EID[21,22]
	Equipment	1st Aspiration system	$6.83x10^{-5}$ p	Modeled from EID sub processes
		2nd Aspiration system	$2.28x10^{-5}$ p	Modeled from EID sub processes
		Scrubber	$1.20x10^{-4}$ p	Modeled from EID sub processes
		Active carbon filter	$3.99x10^{-5}$ p	Modeled from EID sub processes
		Leachate collection tank	$4.91x10^{-5}$ p	Modeled from EID sub processes
	Processes	Sieving of waste from incinerator	0.18 ton	Modeled from EID sub processes
		Leachate treatment	36.14 ton	Modeled from EID sub processes
Outputs	Selected emissions to air[23]	Ammonia	1037.72 g	Simapro airborne emission substance list
		Hydrogen sulfide	3.64 g	Simapro airborne emission substance list
		Benzene	0.78 g	Simapro airborne emission substance list
		Chloroform	0.78 g	Simapro airborne emission substance list
		Toluene	0.78 g	Simapro airborne emission substance list
		Styrene	0.78 g	Simapro airborne emission substance list
		Asbestos fibers[24]	3.98 g	Simapro airborne emission substance list
	Emissions to water	Ammonia	5.23 g	Simapro waterborne emission substance list
End of life	Waste treatment	Long term groundwater emissions	150 ton	EID[25]

[1]This material that needs indeed to be disposed in landfills was used also as the covering of the landfill itself. This amount is of course comprised in the total amount of waste disposed, but its use as the final covering layer makes unnecessary the use of a certain amount of what conventionally employed for this purpose (i.e. sand).
[2]The EID process used was: Sand {RoW}| gravel and quarry operation.
[3]The EID process used was: Occupation, construction site.
[4]The EID process used was: Occupation, dump site.
[5]The EID process used was: Occupation, traffic area.
[6]The EID process used was: Transformation, to construction site.
[7]The EID process used was: Transformation, to dump site, residual material landfill.

[8]The EID process used was: Transformation, to traffic area.
[9]The EID process used was: Transformation, from pasture, man made.
[10]The EID process used was: Sodium hypochlorite, without water, in 15% solution state {RoW}| sodium hypochlorite production, product in 15% solution state.
[11]The EID process used was: Sulfuric acid {RoW}| production.
[12]The EID process used was: Sodium tripolyphosphate {RER}| production.
[13]The EID process used was: Ammonium carbonate {RER}| production.
[14]The EID process used was: Sand {RoW}| gravel and quarry operation.
[15]The EID process used was: Clay {RoW}| clay pit operation.
[16]The EID process used was: Bentonite {RoW}| quarry operation.
[17]The EID process used was: Polypropylene, granulate {RER}| production.
[18]The EID process used was: Polyethylene, high density, granulate {RoW}| production.
[19]The EID process used was: Spinning, bast fibre {RoW}| processing.
[20]The EID process used was: Weaving, bast fibre {RoW}| processing.
[21]The EID process used was: Transport, freight, lorry 16-32 metric ton, EURO6 {RoW}| transport, freight, lorry 16-32 metric ton, EURO6.
[22]An average distance of 100 km was considered for the road freight transport by lorry of materials.
[23]All the emission values reported are primary data, thus directly collected by the operators of the landfill.
[24] This emission was originally referred with the general term "aero dispersed fibrous material" thus potentially containing asbestos, which was however not separately quantified. From this primary data the authors decided to use a precautionary approach and consider all that material as asbestos fibers.
The presence of asbestos fibers release during the disposal of ACWs in the landfill for hazardous waste should not surprise, since although the material was opportunely enclosed according with the Italian legislation, the natural degradation and or accidental damage of both the material and the packaging during the whole landfill lifetime, needs to be considered and accounted for.
[25]The EID process used was: Redmud from bauxite digestion {RoW}| treatment of residual material landfill. The process was modified in order to consider only the long-term emissions in groundwater.

Table S6. Physicochemical properties, degradation rates and toxicity parameters used to model asbestos fibers in USEtox.

**Chemical and physical properties**		**Degradation rates**		**Ecotoxicity**	
Molecular weight[1]	1.62×10^{8} g/mol	in air, K_{degA}[5]	1.0×10^{-20} s^{-1}	logHC50[8]	0.79
Octanol-water partition coefficient, K_{ow}[2]	1.03×10^{30}	in water, K_{degW}[6]	4.5×10^{-8} s^{-1}		
Water solubility at 25°C[2]	1.92×10^{-5} mg/l	in sediment, K_{degSD}[7]	5.0×10^{-9} s^{-1}		
Organic-carbon partition coefficient, K_{oc}[3]	2.58×10^{24} l/kg	in soil, K_{degS}[7]	2.25×10^{-8} s^{-1}	**Human toxicity**	
Henry law coefficient at 25°C, K_H25C[4]	1.0×10^{-20} $Pa \cdot m^3/mol$			$ED50_{ingestion, non\ cancer}$	0[9]
Vapour pressure, P_{vap}[4]	0 Pa			$ED50_{inhalation, non\ cancer}$	0[9]
				$ED50_{ingestion, cancer}$	0[9]
				$ED50_{inhalation, cancer}$[10]	6.5×10^{-3} (kg_{intake}/lifetime)

[1]The molecular weight of multi-walled carbon nanotubes possessing a diameter of 13 nm, a length of 1μm and 27 layers was used, similarly to what considered in the study by Rodriguez-Garcia et al. [6]. This value was calculated according to the procedure recommended in [7].
[2]The K_{ow} as well as the water solubility at 25°C values of multi-walled carbon nanotubes were used. These values were calculated according to [8] by considering the components of the chiral vector, n and m respectively equal to 9 and 0.
[3]The organic-carbon partition coefficient was calculated as recommended by the USEtox User's manual [9], based the formula: $K_{OC} = 1.26 \times K_{OW}^{0.81}$.
[4]The K_H25C value for asbestos fibers was considered the same as the value for multi-walled carbon nanotubes [6], for which the vapour pressure was set to make the Henry constant equal to 1×10^{-20}, as USEtox considers for metals [9], since the vapour pressure of carbon at standard conditions is virtually none [10].
[5]The degradation rate of asbestos fibers in air was considered the same as the one considered for carbon nanotubes in [6], where the value was set to 1×10^{-20} as USEtox does for metals [9], since carbon nanotubes are not degraded by free-radicals, but they induce the formation of free radicals, thus the oxidation of other substances [11].
[6]The degradation rate of asbestos fibers in water was considered the same as the one considered for carbon nanotubes in [6], where the value was calculated assuming their recalcitrant character (according to [12]). Therefore, by using the "recalcitrant" output of the Biowin3 model of EPI Suite [13], the recommended (please refer to page 60 of the USEtox User's manual [9]) half-life and biodegradation rate values of 180 days and 4.5×10^{-8} s^{-1} were considered. In order to extrapolate the biodegradation rate for water, a division factor of 1 is suggested by the EPI Suite [9, 13].
[7]In order to extrapolate biodegradation rates for sediment and soil, the division factors of 9 and 2 were used, as suggested by the EPI Suite [9, 13].
[8]The aquatic ecotoxicity value was calculated according to what performed by Rodriguez-Garcia et al. [6] for multi walled carbon nanotubes. The acute toxicity EC_{50} values for the three species *Chlorella vulgaris, Daphnia magna* and *Danio rerio* are those reported in [14], [15] and [16] respectively. Particularly, the value of 1.8 mg/L, i.e. referred to pristine and well dispersed carbon nanotubes was considered as the EC_{50} value for *Chlorella vulgaris*. As concern the

EC_{50} value for *Daphnia magna* and *Danio rerio*, the values of 8.723 mg/L and 120 mg/L respectively were used. Since, as recommended in the USEtox User's manual [9], the corresponding chronic EC_{50} values need to be preferred, an acute-to-chronic extrapolation factor of 2 was applied [17]. The log HC50 value required by USEtox for the calculation of the ecotoxicological effect factor was then calculated by the formula $\log HC50 = \frac{1}{n_s} \cdot \sum_s logEC50_s$, where n_s is the number of the species (s) [9].
[9]The zero value simply indicates that this toxicity effect was not considered in the present study, which is instead focused on the carcinogenic effect related to inhalation of asbestos fibers.
[10]The carcinogenic effect for humans related to inhalation of asbestos fibers, i.e. the $ED50_{inhalation,\ cancer}$ value, was calculated according to the formula $ED50_{inhalation,cancer}(\frac{kg_{intake}}{lifetime}) = \frac{ED50_{a,t,j} \cdot BW \cdot LT \cdot N}{10^6}$, where $ED50_{a,t,j}$ is the daily dose for animal a (humans in this case) and time duration t (chronic exposition is considered in this case) per kg body weight that causes a disease probability of 50% for exposure route j (inhalation route is considered in this case), BW is the average body weight of humans (i.e. 70 kg), LT is the average lifetime of humans (i.e. 70 years) and N is the number of days per year (i.e. 365 days/year) [9]. The $ED50_{a,t,j}$ value for asbestos fibers was calculated according to the formula $ED50_{a,t,j} = \frac{1}{q_{a,t,j}} \cdot AF_q$, where $q_{a,t,j}$ is the carcinogenic, low dose, slope factor for animal a (humans in this case) and time duration t (chronic exposition is considered in this case) for exposure route j (i.e. inhalation, $kg \cdot day \cdot mg^{-1}$), and AF_q is the extrapolation factor for 1/q to ED50, which is a value of 0.8 [18]. The asbestos carcinogenic slope factor for humans, considering a chronic inhalation exposure, q, was considered equal to 2.2×10^2 $(mg/kg \cdot day)^{-1}$ [19].

Table S7. Detailed quantitative LCIA results for the ACMs management solution comprising the thermal inertisation end of life scenario of 150 ton of ACW.

Impact category	Unit	Mapping the presence/conservation status of ACMs and prioritizing interventions	Encapsulation, removal and storage of ACWs	Thermal inertisation treatment of ACWs	Total
Human toxicity, cancer	cases	$6.20x10^{-8}$	$2.03x10^{-3}$	$1.30x10^{-3}$	$3.33x10^{-3}$
Human toxicity, non-cancer	cases	$1.24x10^{-7}$	$7.41x10^{-4}$	$3.74x10^{-3}$	$4.48x10^{-3}$
Freshwater ecotoxicity	$PAF \cdot m^3 \cdot day$	$5.09x10^{3}$	$6.13x10^{7}$	$1.90x10^{9}$	$1.96x10^{9}$

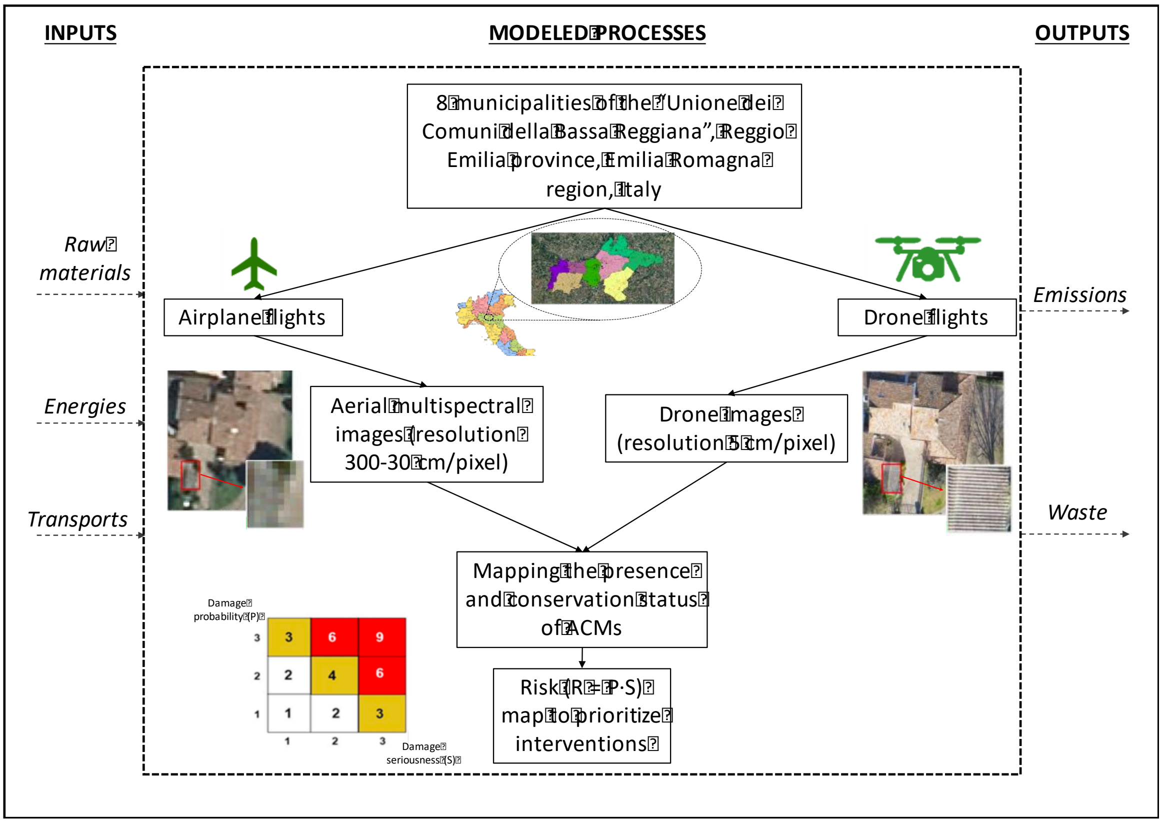

Figure S1. Flowchart showing the system boundaries considered in the LCA of the ACMs mapping and prioritizing intervention activities.

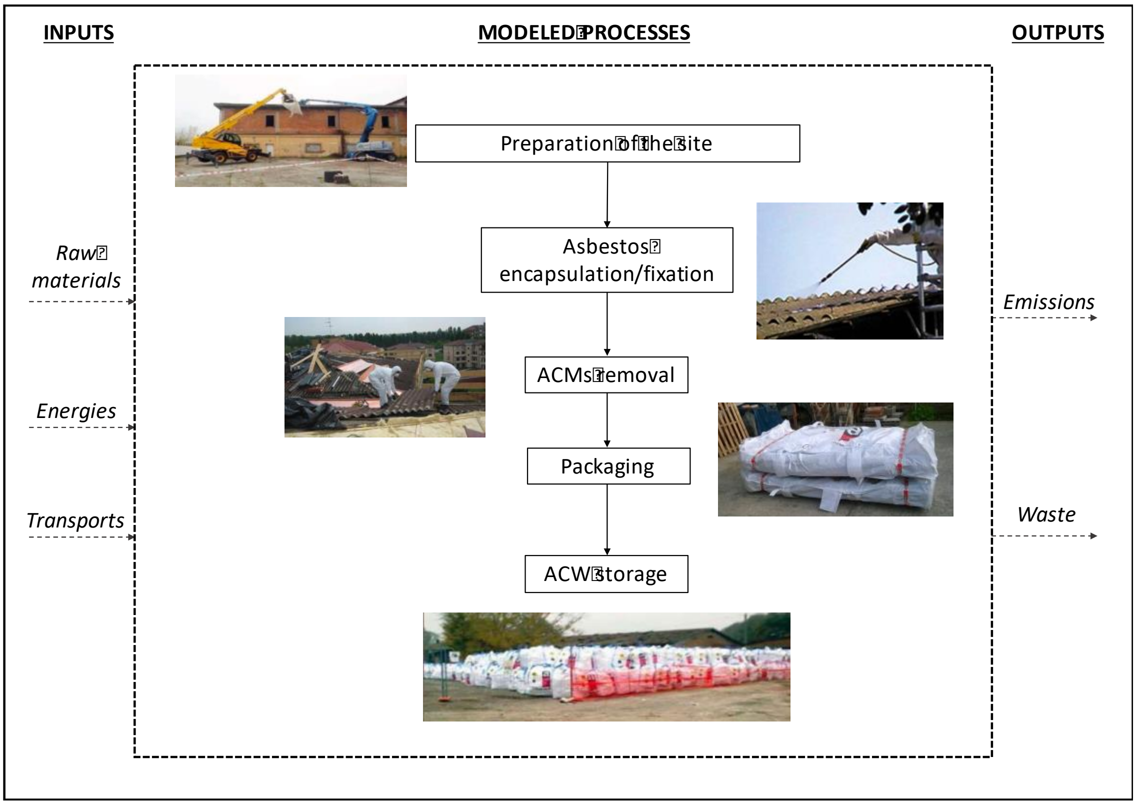

Figure S2. Flowchart showing the system boundaries considered in the LCA of the ACMs safe encapsulation, removal, packaging and preliminary storage of ACWs before their end of life scenario.

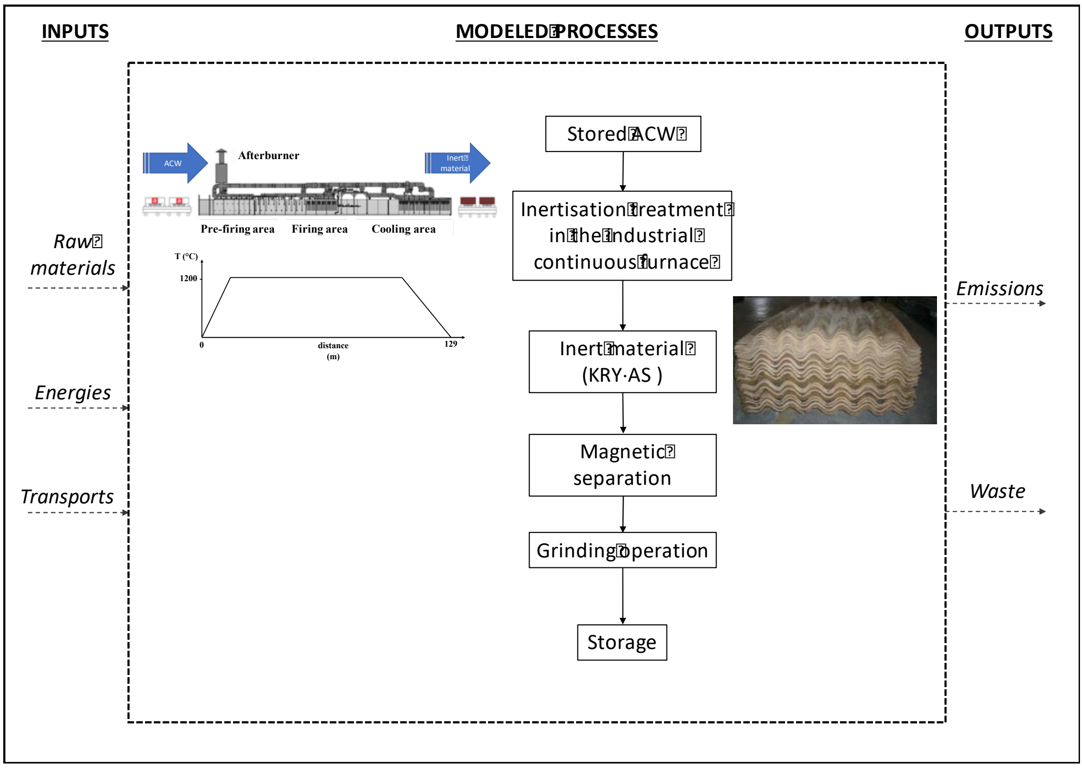

Figure S3. Flowchart showing the system boundaries considered in the LCA of the ACW thermal inertisation treatment by an industrial continuous tunnel kiln.

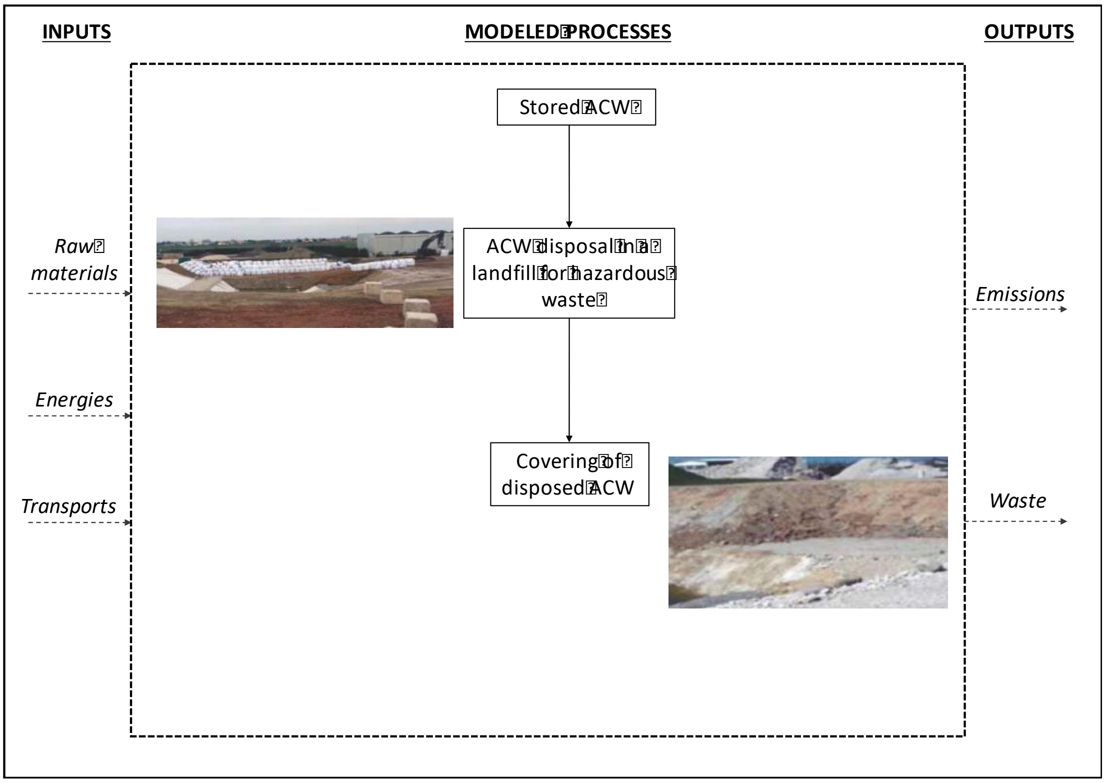

Figure S4. Flowchart showing the system boundaries considered in the LCA of the ACW disposal in a dedicated landfill for hazardous wastes.

APPENDIX A

Analysis of the particulate found in the emissions released during the thermal inertisation process of asbestos containing waste.

No asbestos fibers were released during the innovative thermal inertisation treatment proposed for asbestos containing waste, as recently demonstrated by Tomassetti et al. [5]. Indeed, filters collected during those experiments were analysed using scanning electron microscopy and x-ray microanalysis (SEM-EDS) to assess whether asbestos fibers were emitted in the atmosphere during the thermal degradation of asbestos containing wastes.

The SEM-EDS analysis confirmed the presence of typical microstructures resulting from chrysotile recrystallization process. The crystal habit of the pristine fibers is completely transformed after the thermal treatment, showing an inter-growth of newly-formed crystals with sub-spherical habit. The EDS spectra (not reported here) confirm the presence of Ca- and Mg-silicates produced after the thermal process. The gallery of images below (**Figures S5-S12**) reports selected SEM micrographs of the two samples subjected to the thermal process described in Tomassetti et al. [5], specifically sample C and sample CP. Sample C was commercial asbestos cement, chosen because it is the most prevalent kind of asbestos containing waste. Samples C consisted in a stack of 8 asbestos cement sheets. Sample CP was a block of asbestos cement with polymers. It was chosen because it is also a quite common kind of asbestos containing waste and a significant emission of pollutants was expected, due to the presence of organic polymers [5].

A very few fibers were detected on the particulate collected on the filters. None of the particles have been classified as asbestos. Most of the detected fibers are indeed pseudo fibers formed through the pseudomorphosis phenomenon, thus consisting in truth in aggregates of small newly formed crystals (resembling only apparently the fibrous crystal habit) that intimately transformed the pristine fibers.

The other fibers are exotic, being ceramic refractory fibers released from the wool medium used in the experiment to provide a better insulation of the furnace and pipes. Although these exotic fibers,

could be potentially included in the Fiber Pathogenicity Paradigm (FPP) [20], this is however a not quantifiable rarity, that in all likelihood could be avoided by exploring alternative refractory materials.

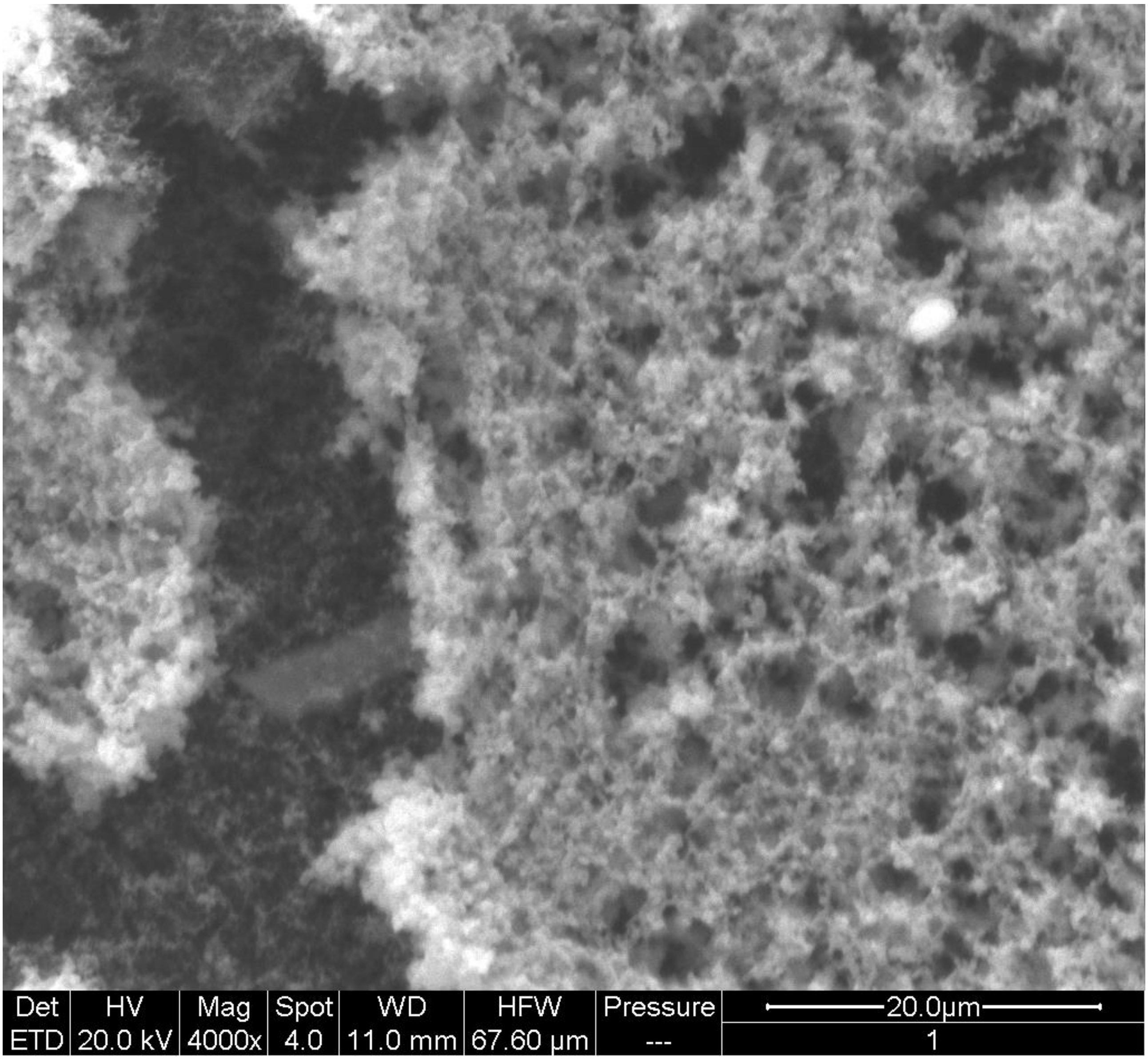

Figure S5. Sample C – The particulate matrix.

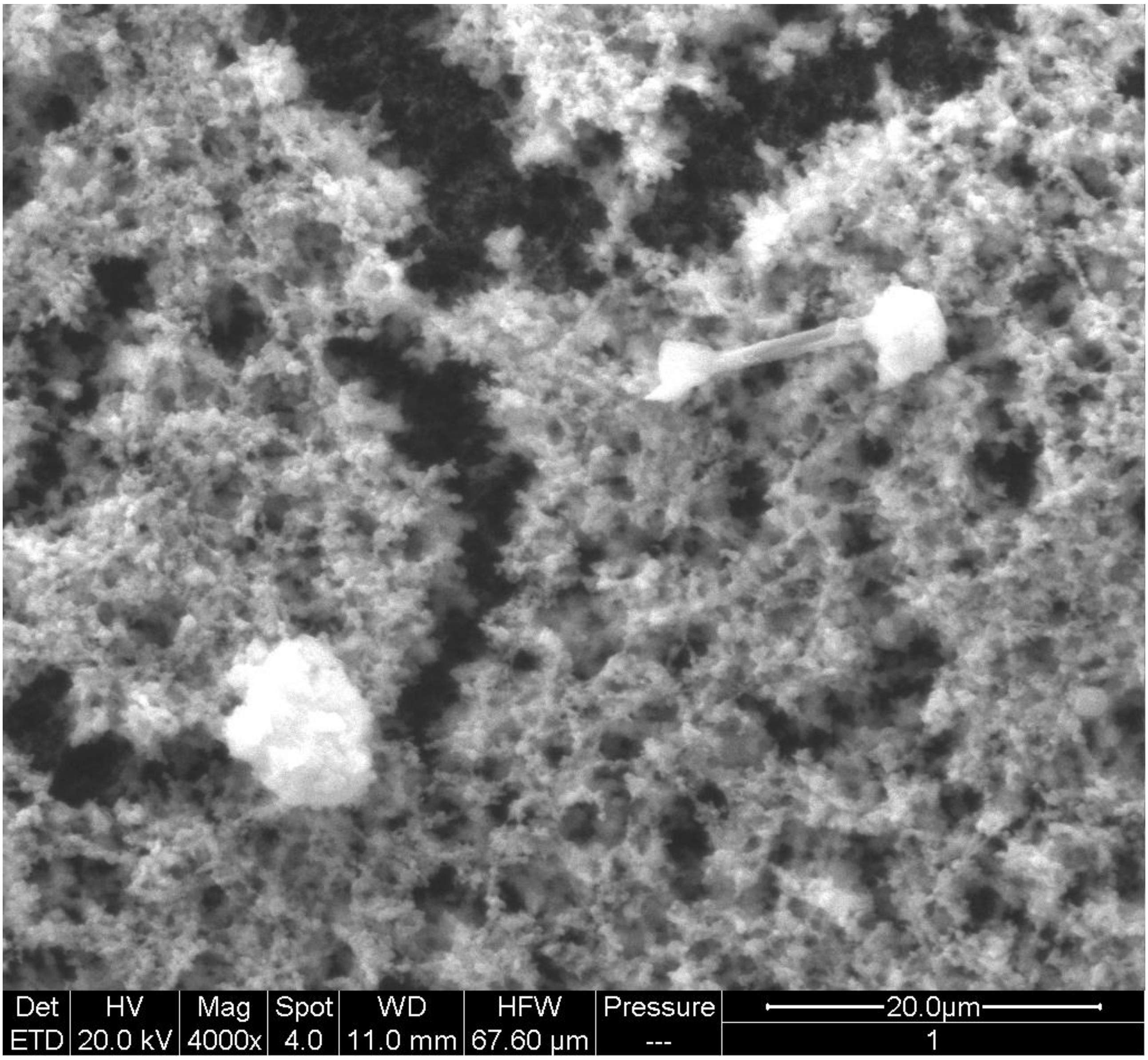

Figure S6. Sample C – The particulate matrix.

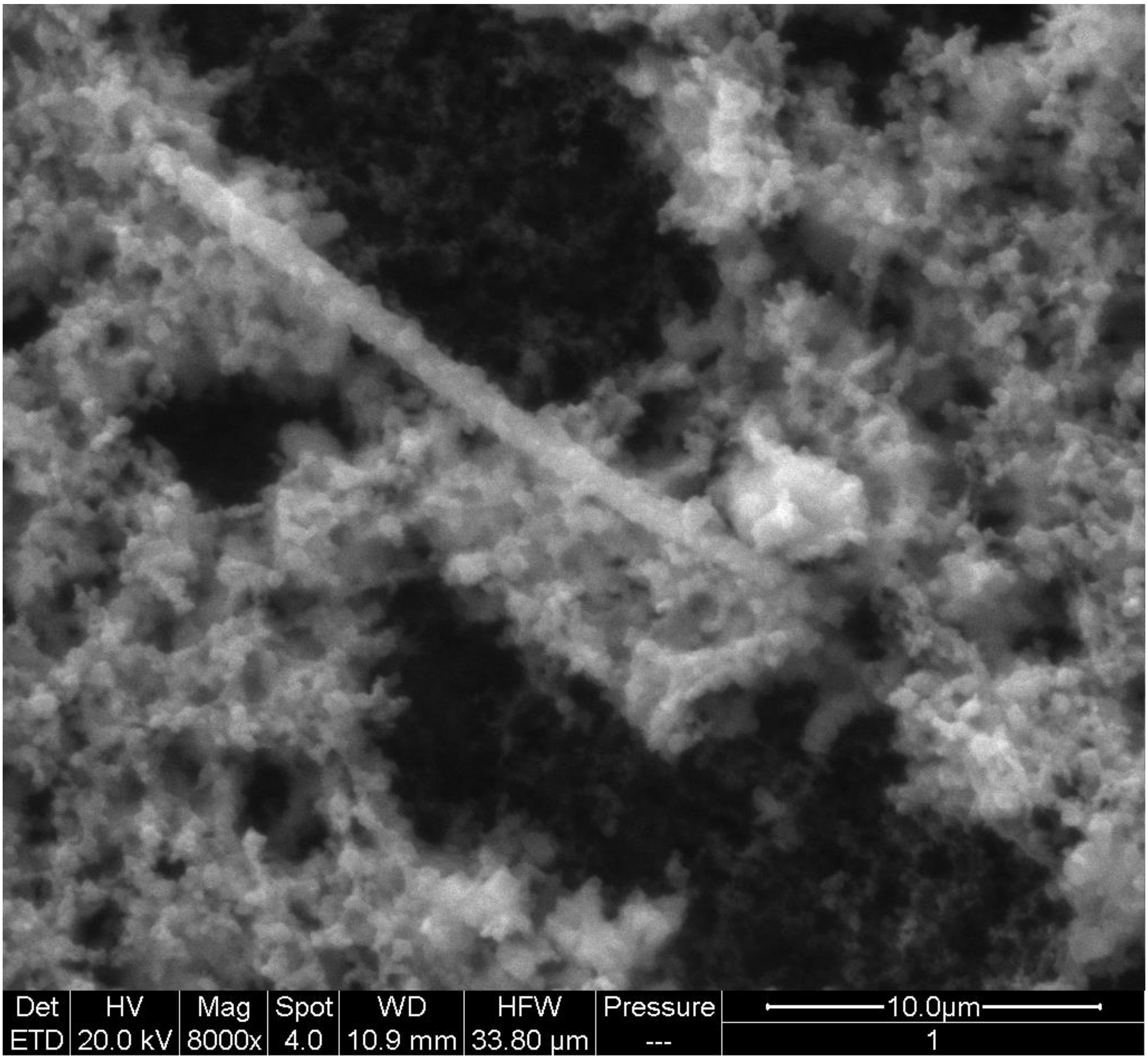

Figure S7. Sample C – Particulate matrix with a totally recrystallized pristine asbestos fibre.

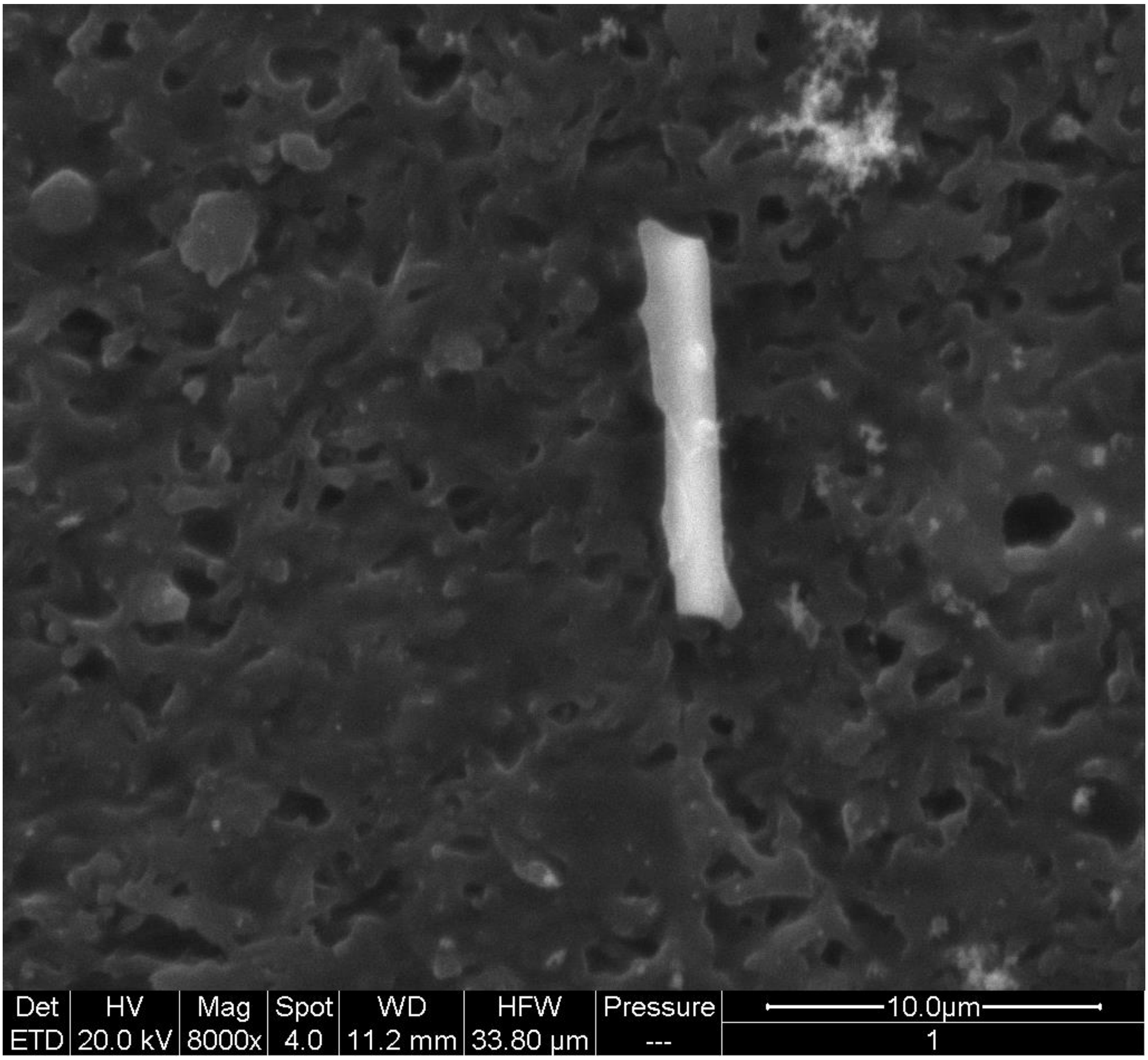

Figure S8. Sample C – A fragment of exotic synthetic ceramic fibre.

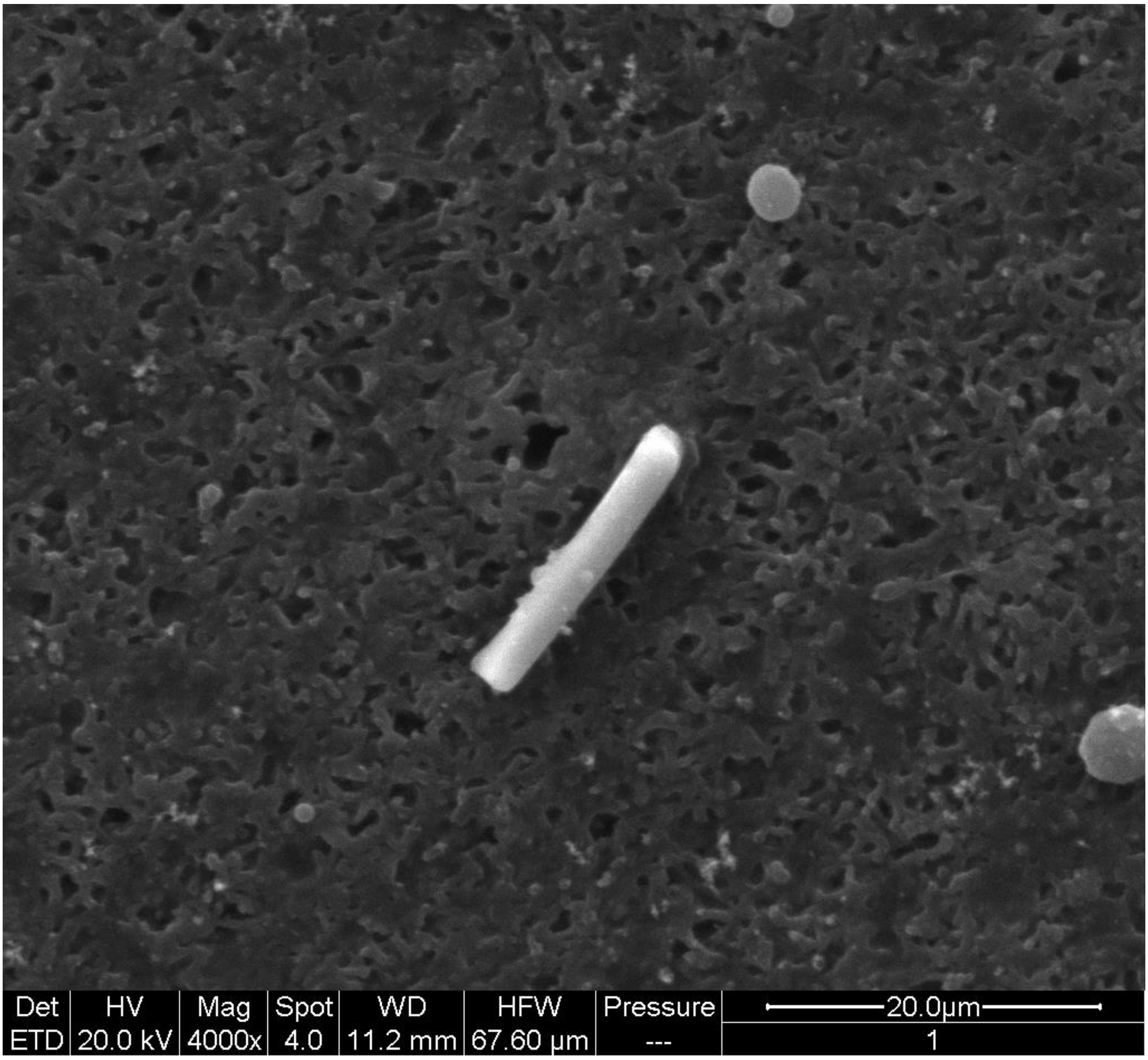

Figure S9. Sample CP – A short exotic synthetic ceramic fibre.

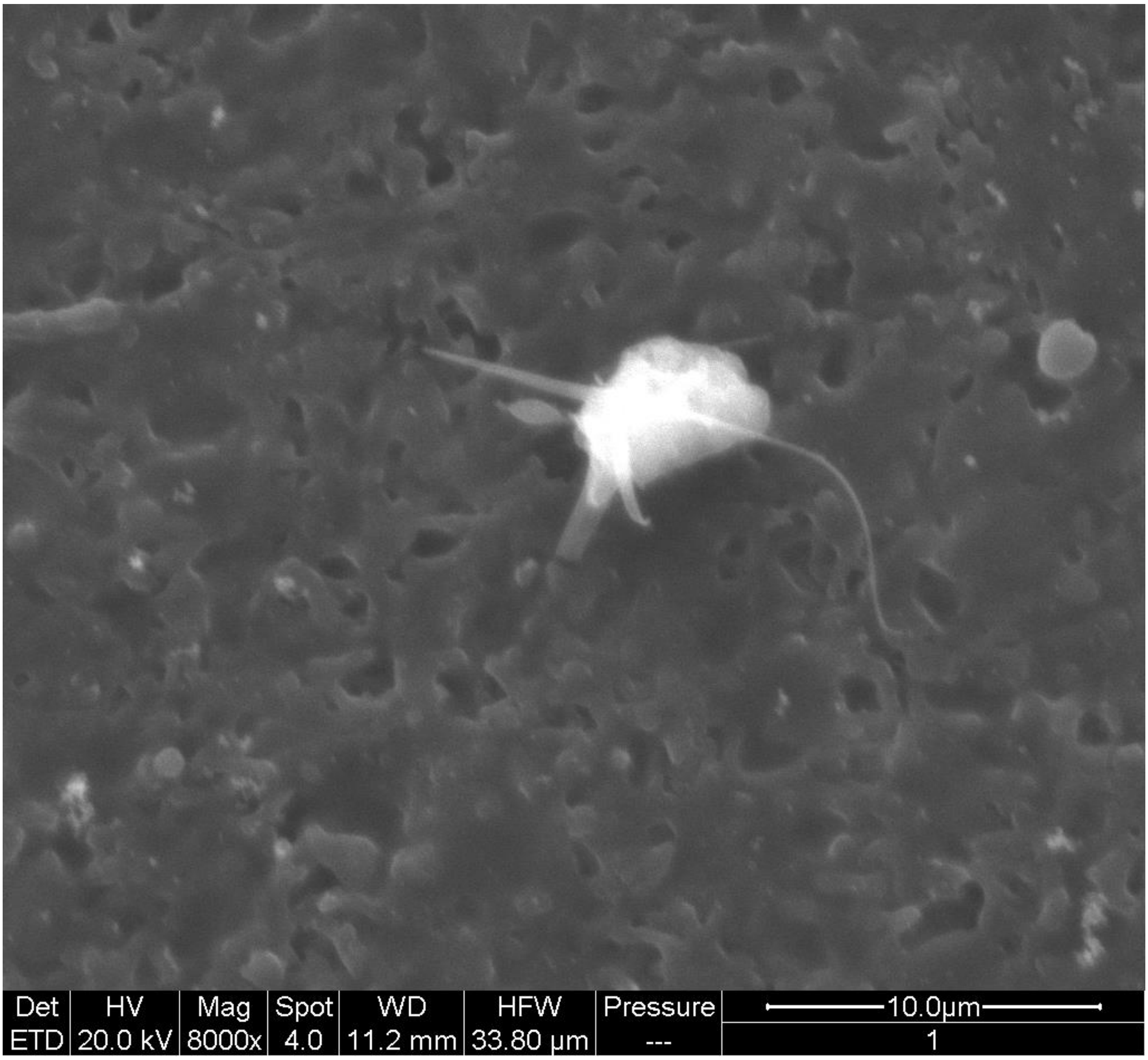

Figure S10. Sample CP – Particulate cluster embedding synthetic exotic ceramic fibres.

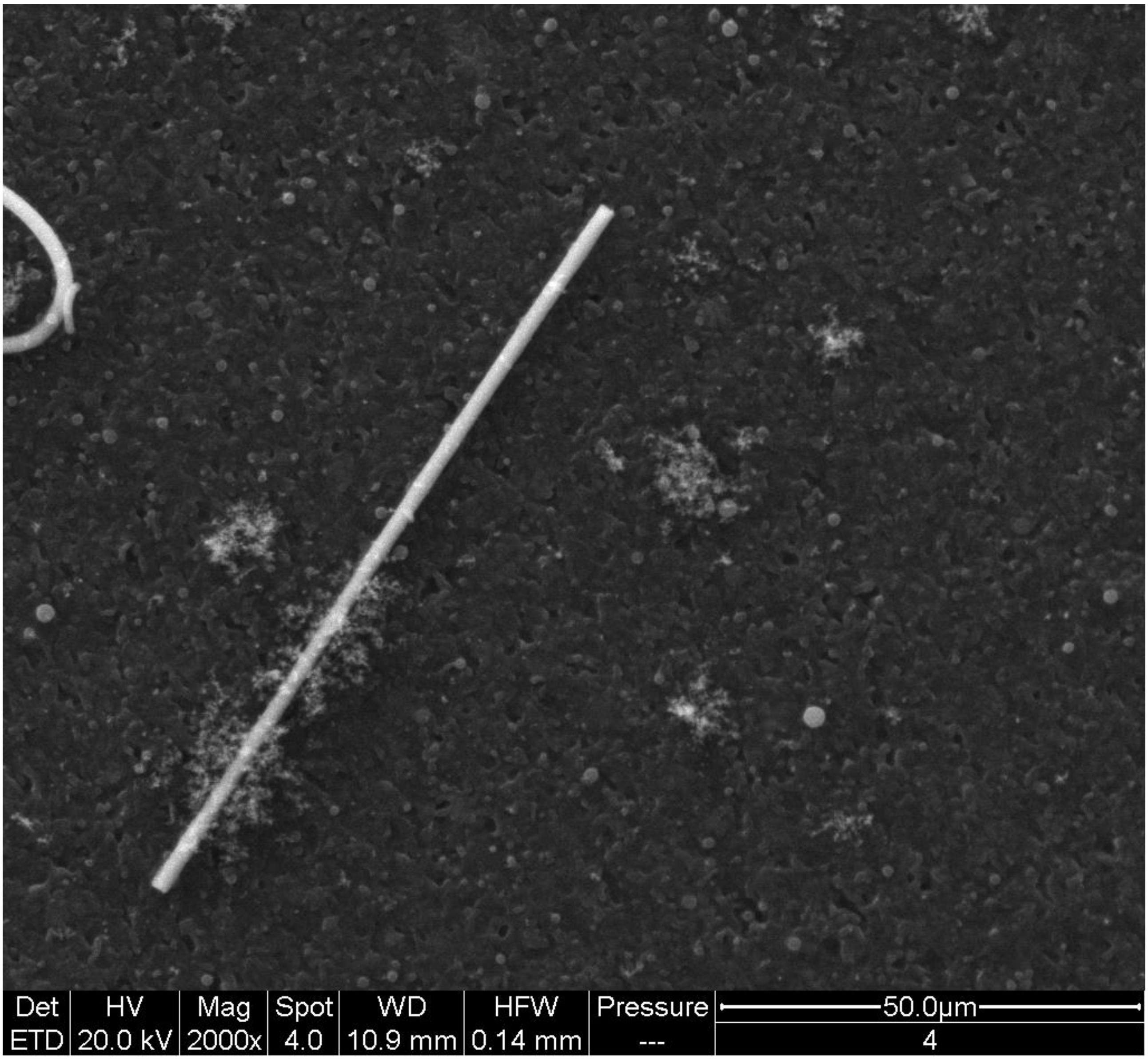

Figure S11. Sample CP – Particulate matrix with a long exotic synthetic ceramic fibre.

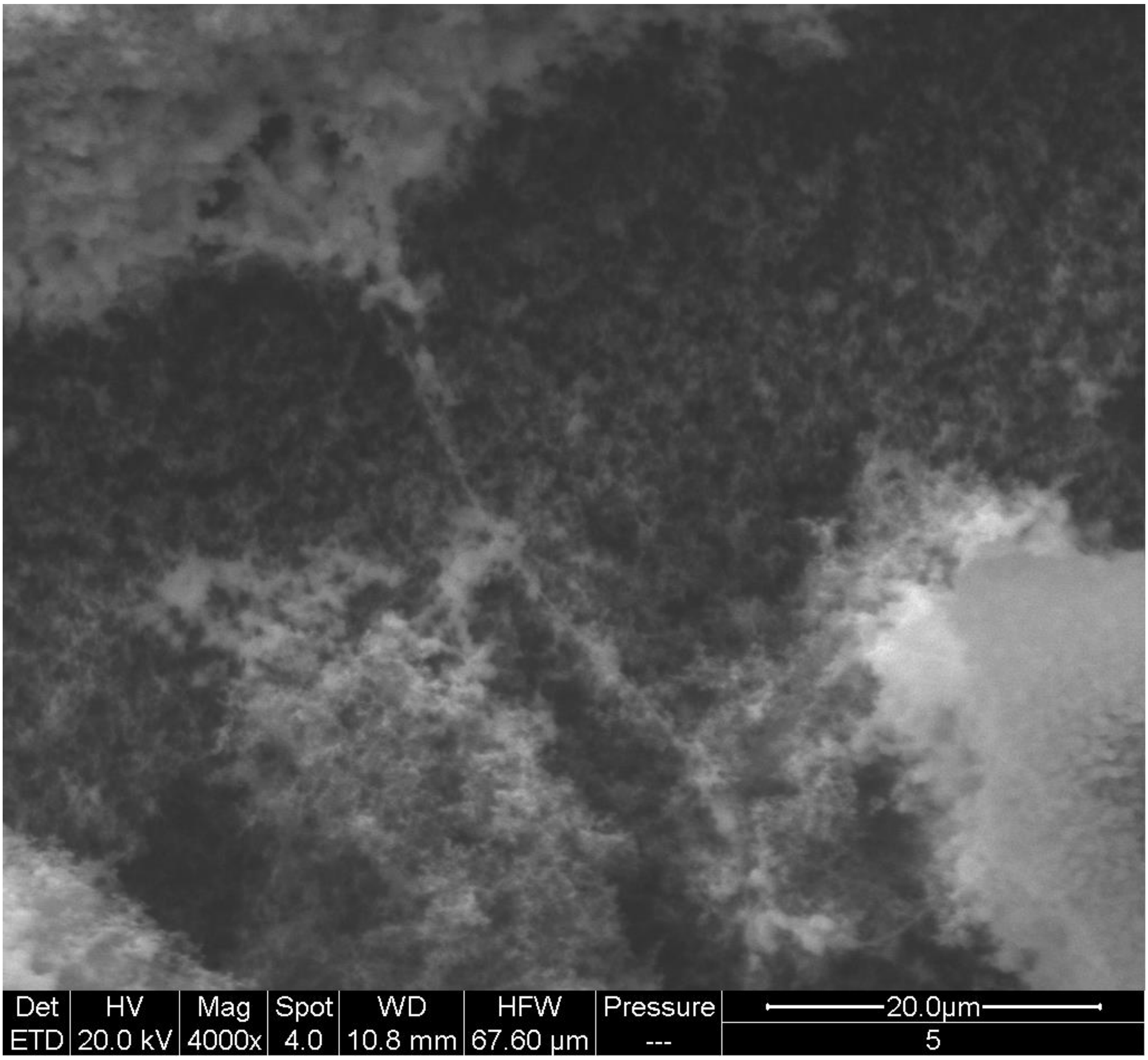

Figure S12. Sample CP – Particulate matrix with a long exotic synthetic ceramic fibre.

APPENDIX B

Considerations and assumptions made for the calculation of asbestos fibers released as a consequence of the not removal at all of the 58 millions m^2 of asbestos containing materials requiring intervention still present in Italy.

A recent Italian survey highlighted the presence of *ca*. $58x10^6$ m^2 of asbestos containing materials in the coverings of Italian public and private buildings as well as industrial sites [21].

The average weight of ACMs was considered 17 kg/m^2, while the average composition of asbestos fibers in ACMs was considered 10 wt%, similarly to what considered in the whole study, in full agreement with literature [3].

Therefore, the asbestos fibers contained in $58x10^6$ $m^2 \cdot 17$ kg/m2 /1000 = 986000 ton of ACMs are 0.10·986000 = 98600 ton.

The existing coverings in the year 2021 have a minimum age of 29 years, since the use of asbestos was banned in Italy in 1992.

It has been supposed that the 50% of the coverings dates back to 1980 while the remaining 50% dates back to 1992.

It has been supposed that the structure of asbestos containing materials starts degrading after 30 years, so that the ACMs set down in 1980 started their degradation in 2010, while those set down in 1992 will start their degradation in 2022.

It has been supposed a progressive degradation of ACMs with the latter being completed in 50 years, with the complete release of asbestos fibers.

Thus, by considering the 2030 scenario:

- the percentage of asbestos fibers released from the coverings set down in 1980 will be (2030-2010)/(2060-2010)·100 = 40%
- the percentage of asbestos fibers released from the coverings set down in 1992 will be (2030-2022)/(2072-2022)·100 = 16%

REFERENCES

[1] Italian Ministerial Decree, August 20th, 1999. Upgrade of the legislation and technical methodologies for remediation interventions, comprising those for asbestos inertization planned by article 5, subsection 1, letter f of the Italian law n°257 of March 27th, 1992, comprising the legislation related to the termination of asbestos use. Italian Gazzetta Ufficiale October 22nd, 1999, n° 249.
[2] Italian Ministerial Decree, September 6th, 1994. Legislation and technical methodologies application of article 6, subsection 3, and article 12, subsection 2 of the Italian law n° 257 of March 27th, 1992, on to the termination of asbestos use. Italian Gazzetta Ufficiale, September 20th, 1994, n° 220.
[3] Ervik, T.; Hammer, S. E.; Graff, P. Mobilization of asbestos fibers by weathering of a corrugated asbestos cement roof. *J. Occup. Environ. Hygiene* **2021**, DOI: 10.1080/15459624.2020.1867730.
[4] https://www.sabar.it/.
[5] Tomassetti, L.; Di Giuseppe, D.; Zoboli, A.; Paolini, V.; Torre, M.; Paris, E.; Guerriero, E.; Petracchini, F.; Gualtieri, A. F. Emission of fibres and atmospheric pollutants from the thermal treatment of asbestos containing waste (ACW). *J. Clean. Prod*. **2020**, 268, 122179.
[6] Rodriguez-Garcia, G.; Zimmermann, B.; Weil, M. Nanotoxicity and Life Cycle Assessment: First attempt towards the determination of characterization factors for carbon nanotubes. IOP Conf. Ser. Mater. Sci. Eng. 2014, 64, 12029.
[7] Laurent, C.; Flahaut, E.; Peigney, A. The weight and density of carbon nanotubes versus the number of walls and diameter. *Carbon* **2010**, 48, 2989-2999.
[8] Toropov, A. A.; Leszczynska, D.; Leszczynski, J. Predicting water solubility and octanol water partition coefficient for carbon nanotubes based on the chiral vector. *Comput. Biol. Chem*. **2007**, 31, 127-128.
[9] Huijbregts, M.; Hauschild, M.; Jolliet, O.; Margni, M.; McKone, T.; Rosenbaum, R. K.; van de Meent, D. USEtox™ User manual, version 1.01, 2010.
[10] Leider, H. R.; Krikorian, O. H.; Young, D. A. Thermodynamic properties of carbon up to the critical point. *Carbon* **1973**, 11, 555-563.
[11] Shvedova, A. A.; Castra nova, V.; Kisin, E. R.; Schwegler-Berry, D.; Murray, A. R.; Gandelsman, V. Z.; Maynard, A.; Baron, P. Exposure to carbon nanotube material: assessment of nanotube cytotoxicity using human keratinocyte cells. *J. Toxicol. Environ. Health A* **2003**, 66, 1909-1926.
[12] Kümmerer, K.; Menz, J.; Schubert, T.; Thielemans, W. Biodegradability of organic nanoparticles in the aqueous environment. *Chemosphere* **2011**, 82, 1387-1392.
[13] EPI Suite™-Estimation Program Interface, https://www.epa.gov/tsca-screening-tools/epi-suitetm-estimation-program-interface#what.
[14] Schwab, F.; Bucheli, T. D.; Lukhele, L. P.; Magrez, A.; Nowack, B.; Sigg, L.; Knauer, K. Are carbon nanotube effects on green algae caused by shading and agglomeration? *Environ. Sci. Technol*. **2011**, 45, 6136-6144.
[15] Zhu, X.; Zhu, L.; Chen, Y.; Tian, S. Acute toxicities of six manufactured nanomaterial suspensions to *Daphnia magna*. *J. Nanopart. Res*. **2009**, 11, 67-75.
[16] Eckelman, M. J.; Mauter, M. S.; Isaacs, J. A.; Elimelech, M. New perspectives on nanomaterial aquatic ecotoxicity: Production impacts exceed direct exposure impacts for carbon nanotubes. *Environ. Sci. Technol.* **2012**, 46, 2902-2910.
[17] Rosenbaum, R. K.; Bachmann, T. M.; Gold, L. S.; Huijbregts, M. A. J.; Jolliet, O.; Juraske, R.; Koehler, A.; Larsen, H. F.; MacLeod, M.; Margni, M.; McKone, T. E.; Payet, J.; Schuhmacher, M.; Van de Meent, D.; Hauschild, M. Z. Usetox – the UNEP-SETAC toxicity model: recommended characterisation factors for human toxicity and freshwater ecotoxicity in life cycle impact assessment. *Int. J. Life Cycle Assess*. **2008**, 13, 532-546.

[18] Huijbregts, M. A. J.; Rombouts, L. J. A.; Ragas, A. M. J.; Van de Meent, D. Human-toxicological effect and damage factors of carcinogenic and non-carcinogenic chemicals for life cycle impact assessment. *Integ. Environ. Assess. Manag.* **2005**, 1, 181-244.
[19] California Office of Environmental Health Hazard Assessment (OEHHA). Technical Support Document for Cancer Potency Factors, 2009, https://oehha.ca.gov/air/crnr/technical-support-document-cancer-potency-factors-2009.
[20] Donaldson, K.; Murphy, F.; Schinwald A.; Duffin, R.; Poland, C. A. Identifying the pulmonary hazard of high aspect ratio nanoparticles to enable their safety-by-design. Nanomedicine 2011, 6, 143-156.
[21] Marian, N. M.; Giorgetti, G.; Magrini, C.; Capitani, G. C.; Galimberti, L.; Cavallo, A.; Salvini, R.; Vanneschi, C.; Viti, C. From hazardous asbestos containing wastes (ACW) to new secondary raw material through a new sustainable inertization process: A multimethodological mineralogical study. *J. Hazard. Mater.* **2021**, 413, 125419.
